# Catalytic [6 + 3] cycloaddition for the synthesis of medium-sized ring systems: a critical review

**DOI:** 10.1039/d6ra05845k

**Published:** 2026-09-07

**Authors:** Archna, Gobind Kumar, Pooja A. Chawla, Rupesh Kumar, Gaurav Bhargava

**Affiliations:** a Department of Chemistry, I. K. Gujral Punjab Technical University Kapurthala Punjab-144603 India; b University Institute of Pharmaceutical Sciences and Research, Baba Farid University of Health Sciences Faridkot Punjab 151203 India gauravorganic@gmail.com gaurav@ptu.ac.in

## Abstract

Medium-sized rings are important structural synthons in numerous natural products, bioactive molecules, and pharmaceutical products. However, their synthesis remains challenging due to unfavourable entropic factors, transannular strain, and conformational flexibility. Among the available synthetic strategies, the [6 + 3] cycloaddition and formal annulation strategies have emerged as a powerful and atom-economical approach for the construction of nine-membered carbocyclic and heterocyclic, fused, and bridged medium-sized ring frameworks. Since 2000, significant progress has been achieved in the development of catalytic systems that enable efficient and selective [6 + 3] cycloaddition processes under mild conditions. This concise review summarizes recent advances in the [6 + 3] cycloaddition reactions since 2000, with a particular emphasis on the role of transition-metal catalysis, Lewis acid catalysis, and organocatalysts, as well as their substrate scope expansion and stereocontrol strategies in the [6 + 3] cycloadditions.

## Abstract 2

Catalytic [6 + 3] cycloaddition are a versatile platform for the concise synthesis of functionalized medium-sized ring systems, with promising applications in natural-product synthesis, medicinal chemistry, and the development of complex molecular architectures.

## Graphical abstract

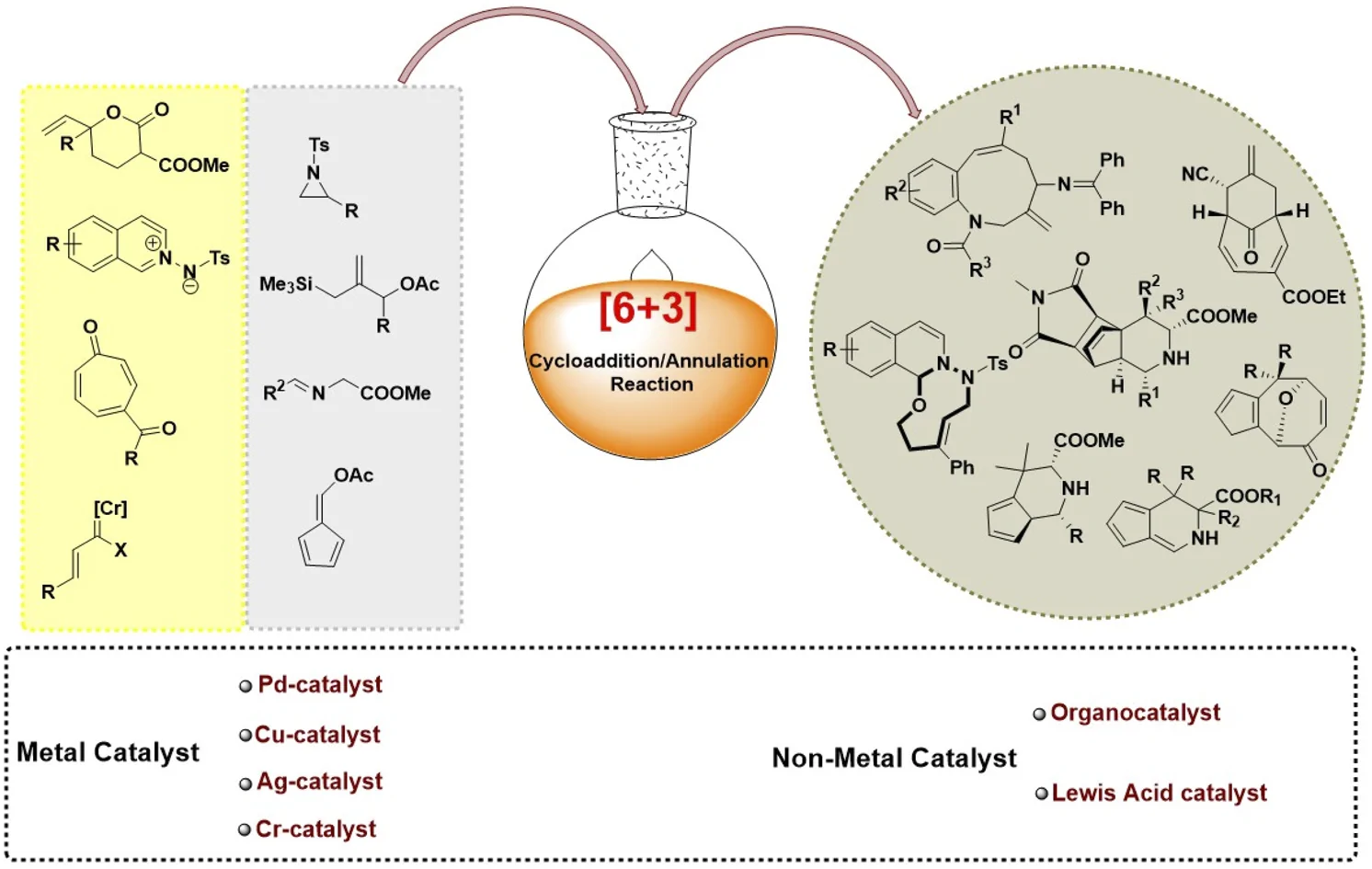

## 1. Introduction

Medium-sized ring systems are prevalent structural motifs in natural products, pharmaceuticals, and advanced functional materials, where their unique conformational properties often translate to valuable biological and physicochemical functions.^1–4^ Among these, nine-membered frameworks are particularly attractive yet synthetically challenging due to unfavourable entropic barriers, transannular interactions, and conformational flexibility that complicate ring construction by conventional methods.^5^ To address this challenge, cycloaddition reactions (CRs) are widely utilized in organic synthesis due to their ability to rapidly generate cyclic structures with high atom economy and excellent stereochemical control.^6^ The foundations of cycloaddition chemistry date back to the early 20th century, most notably with the development of the Diels–Alder reaction, a [4 + 2] cycloaddition that remains a keystone methodology for the synthesis of six-membered ring systems.^7,8^

**Fig. 1:**
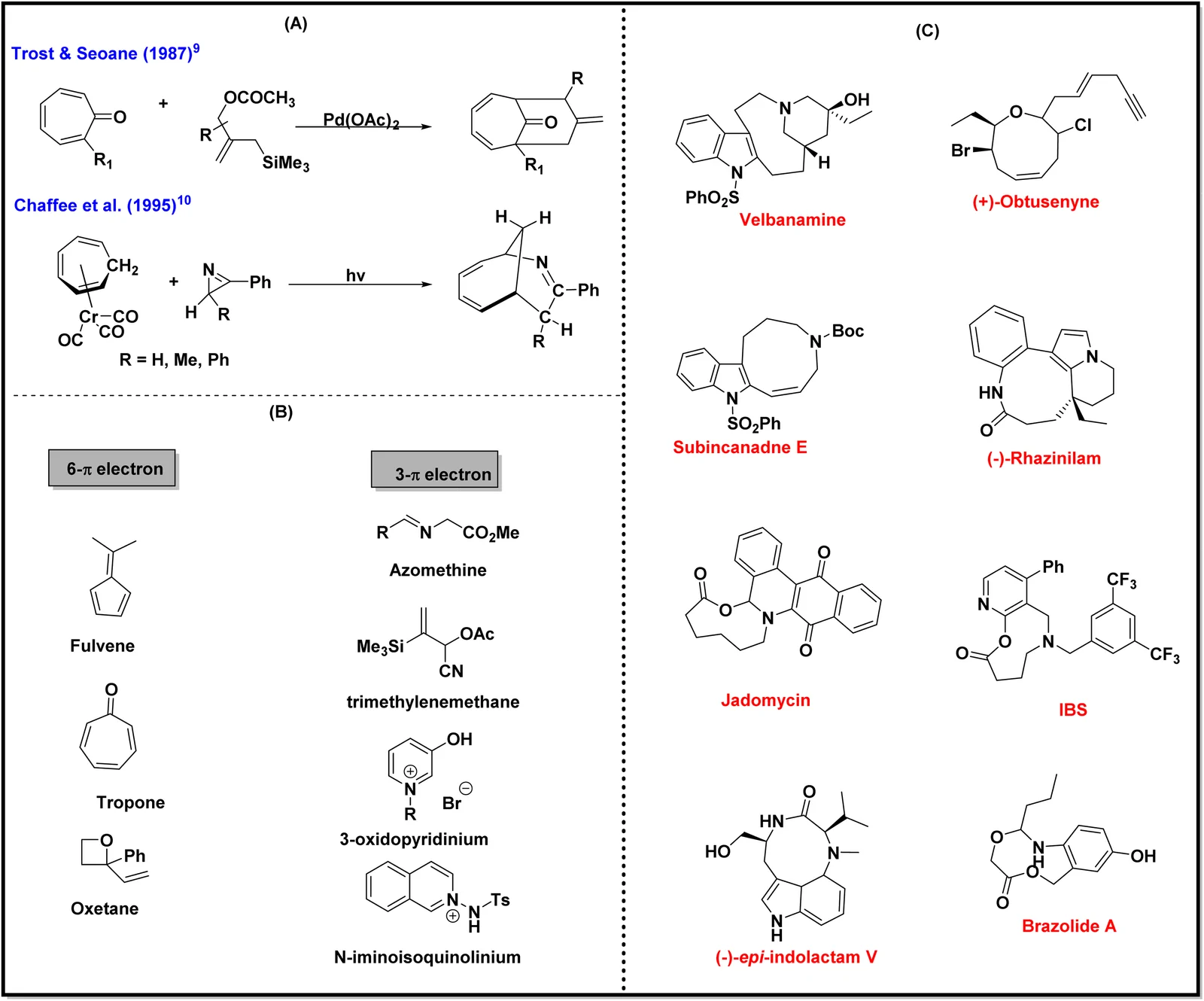
(A) Background of [6 + 3] CR,^9,10^ (B) representation of the 6π and 3π electron synthons utilized in [6 + 3] cycloadditions, & (C) selected natural and synthetic bio-active compounds.^14–17^

Among these, the [6 + 3] cycloaddition has attracted considerable attention as an efficient and convergent strategy for the construction of nine-membered carbocyclic and heterocyclic frameworks through the combination of a 6-atom π-component (*e.g.*, 1,3-dienes or related systems) with a 3-atom synthon, such as allylic cations, cyclopropanes, or azomethine ylides (Fig. 1A).^9^ The origins of [6 + 3] cycloaddition chemistry can be traced to the pioneering work of Trost and Seoane in 1987, who reported one of the earliest examples of nine-membered carbocycle synthesis *via* a [6 + 3] cycloaddition strategy (Fig. 1A).^9^ This landmark study established the conceptual foundation for the construction of medium-sized rings using [6 + 3] cycloaddition chemistry. Subsequently, Chaffee and co-workers in 1995 reported a metal-mediated formal [6 + 3] cycloaddition of 2-phenyl-1-azirines with tricarbonyl(cycloheptatriene)chromium(0) under UV irradiation, providing an efficient route to nine-membered nitrogen-containing bicyclic heterocycles (Fig. 1A).^10^ These pioneering studies demonstrated the feasibility of the [6 + 3] cycloaddition for assembling both carbocyclic and heterocyclic medium-sized ring systems, providing a way for the development of more efficient catalytic methodologies.

On the other hand, aza-nine-membered rings are important structural motifs in many natural products and biomolecules, often associated with significant biological activities.^11–13^ As shown in Fig. 1C, alkaloids such as Sabincanadine,^14^ (−)-Rhazinilam^15^ and Velbenamine^16^ have exhibited diverse pharmacological effects, including antitumor, cell-division regulatory, and anti-inflammatory activities. Moreover, diaza, oxacyclic, and aza-oxa- based nine-membered ring motifs have been found in several biologically active compounds, including indolactam, BrazolidineA, and (+)-Obtusenyne.^14–17^

**Fig. 2:**
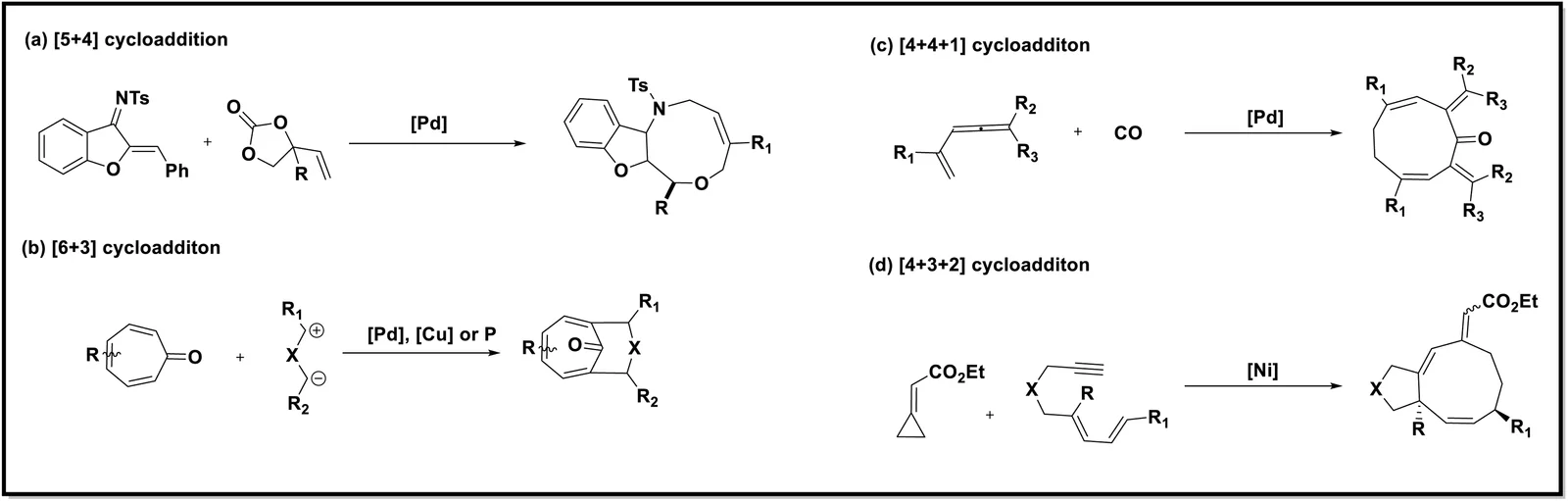
Illustrates different CR (a and b) employed for the construction of a nine-membered ring.^18–25^

As represented in Fig. 2, various atom-economical cycloaddition pathways such as [5 + 4],^18^ [6 + 3],^19–22^ [4 + 4 + 1],^23,24^ and [4 + 3 + 2],^25^ have been explored by different researchers for the construction of the nine-membered functionalized rings.^9,18–20^

Building upon these pioneering studies, significant advances in transition-metal catalysis over the past two decades have substantially expanded the scope, selectivity, and synthetic utility of [6 + 3] cycloaddition reactions.^21^ However, the intrinsic kinetic and thermodynamic challenges associated with forming medium-sized rings, coupled with the need for precise control over chemo-, regio-, and stereoselectivity, have limited the development of general catalytic platforms for this class of reactions.

Catalysts including transition-metal (*e.g.*, rhodium, palladium, nickel), and non-metal complexes (Lewis acids, and organocatalytic systems), have enabled a range of [6 + 3] cycloaddition pathways under milder conditions with improved selectivity. Notably, advances in activation modes such as metal-mediated π-coordination, strain-release from small-ring donors (*e.g.*, vinyl cyclopropanes), and synergistic dual catalysis have broadened substrate scope and enhanced functional-group compatibility. These catalytic strategies have unlocked access to structurally diverse medium-sized ring systems that were previously difficult or inefficient to synthesize.

However, it is important to recognize that the term “[6 + 3] cycloaddition” is often used broadly to describe both true concerted cycloaddition reactions and formal [6 + 3] annulation processes. In true [6 + 3] cycloadditions, bond formation occurs through a single cycloaddition event, whereas formal [6 + 3] annulations generally proceed through stepwise pathways involving metal-mediated π-allyl, zwitterionic, or ionic intermediates. Although both approaches deliver similar nine-membered ring frameworks, their mechanistic features, activation modes, and selectivity-controlling factors are distinct.

Therefore, this concise review provides a critical overview of catalytic [6 + 3] cycloaddition and formal annulation strategies developed since 2000 for the construction of nine-membered carbocyclic, heterocyclic, fused, and bridged medium-sized ring frameworks, with emphasis on catalyst design, mechanistic features, and factors governing chemo-, regio-, and stereoselectivity.

## 2. Catalytic promoted [6 + 3] cycloaddition reactions

### 2.1. Metal-catalyzed [6 + 3] cycloaddition

Metal-catalyzed [6 + 3] cycloaddition reactions provide an efficient strategy for constructing nine-membered carbocyclic and heterocyclic frameworks. The catalytic performance is governed by the metal centre, ligand environment, and substrate electronics, which collectively influence reaction pathways, intermediate stability, and chemo-, regio-, and stereoselectivity. Depending on the metal employed, activation may proceed through π-coordination, oxidative addition, generation of metallo-dipoles, or Lewis acid activation. The following sections critically compare the mechanistic features, catalyst design strategies, and synthetic utility of representative metal-catalyzed [6 + 3] cycloaddition reactions.

#### 2.1.1. Palladium catalyzed [6 + 3] cycloaddition and formal [6 + 3] annulation reactions

Palladium catalysts have gained considerable attention in cycloaddition chemistry due to their versatile coordination ability, tunable electronic properties, and capacity to activate π-components through π-complexation and oxidative addition/reductive elimination pathways. The ability of palladium to stabilize reactive intermediates, such as zwitterionic or dipolar species, and to promote selective C–C and C-heteroatom bond formation makes it an effective catalyst for constructing complex cyclic frameworks under mild reaction conditions.

**Fig. 3:**
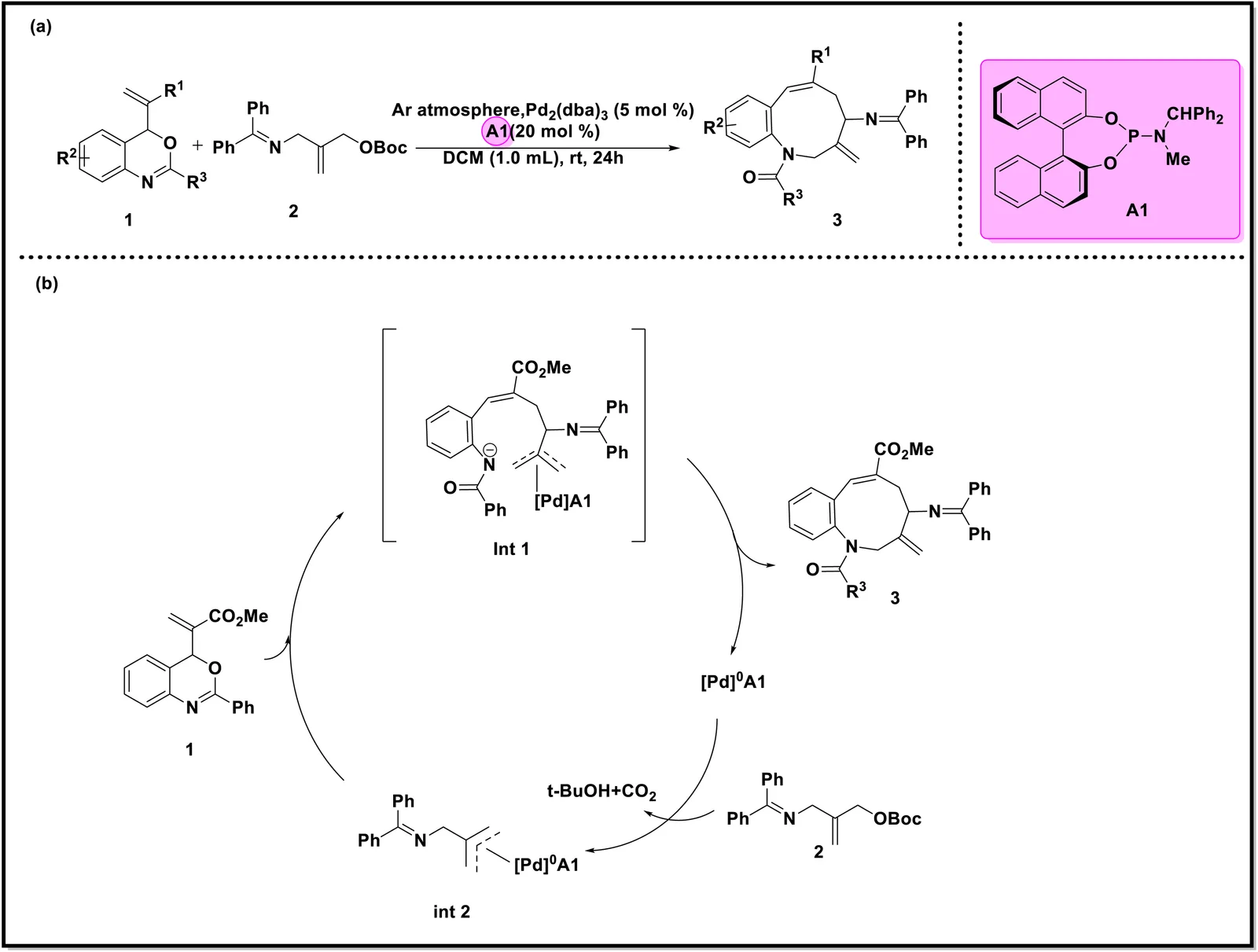
(a) Synthesis of nine-membered heterocycles using Pd catalyst (b) the mechanistic pathway of Pd-catalyzed synthesis of nine-membered heterocycles.^22^

In this context, Chen *et al.*^22^ developed a Pd-catalyzed enantioselective formal (6 + 3) annulation of 2-(4*H*-benzo[*d*][1,3]oxazin-4-yl)acrylates 1 with amino-trimethylenemethane (amino-TMM) 2 to afford nine-membered heterocycles 3. Screening of chiral ligands revealed that ligand A1 was the most effective, delivering the cycloadduct in up to 75% yield and 93% ee. The optimized conditions employed Pd_2_(dba)_3_/A1 in CH_2_Cl_2_ at 0 °C, offered a broad range of 2-(4*H*-benzo[*d*][1,3]oxazin-4-yl)acrylates bearing electron-donating, electron-withdrawing, aromatic, and heteroaromatic substituents reacted smoothly with amino-TMM precursors, furnishing the desired nine-membered heterocycles in 60–75% yields with 77–93% ee, demonstrating excellent functional group tolerance and enantioselectivity (Fig. 3a).

The mechanistic pathway of the reaction revealed that the amino-TMM 2, upon decarboxylation in the presence of the Pd(0)/A1 catalyst, generated a zwitterionic π-allylpalladium intermediate Int 2. Subsequently, the nucleophilic carbon of Int 2 underwent a regioselective Michael addition to the electron-deficient 1, affording zwitterionic intermediate Int 1. The chiral ligand A1 created a sterically defined environment around the palladium centre, directing the approach of the acrylate from the less hindered face and thereby controlling the enantioselectivity of the annulation. Finally, intramolecular allylic substitution of Int 1 furnished the nine-membered (6 + 3) cycloadduct 3 with regeneration of the active Pd(0)/A1 catalyst (Fig. 3b).^22^

**Fig. 4:**
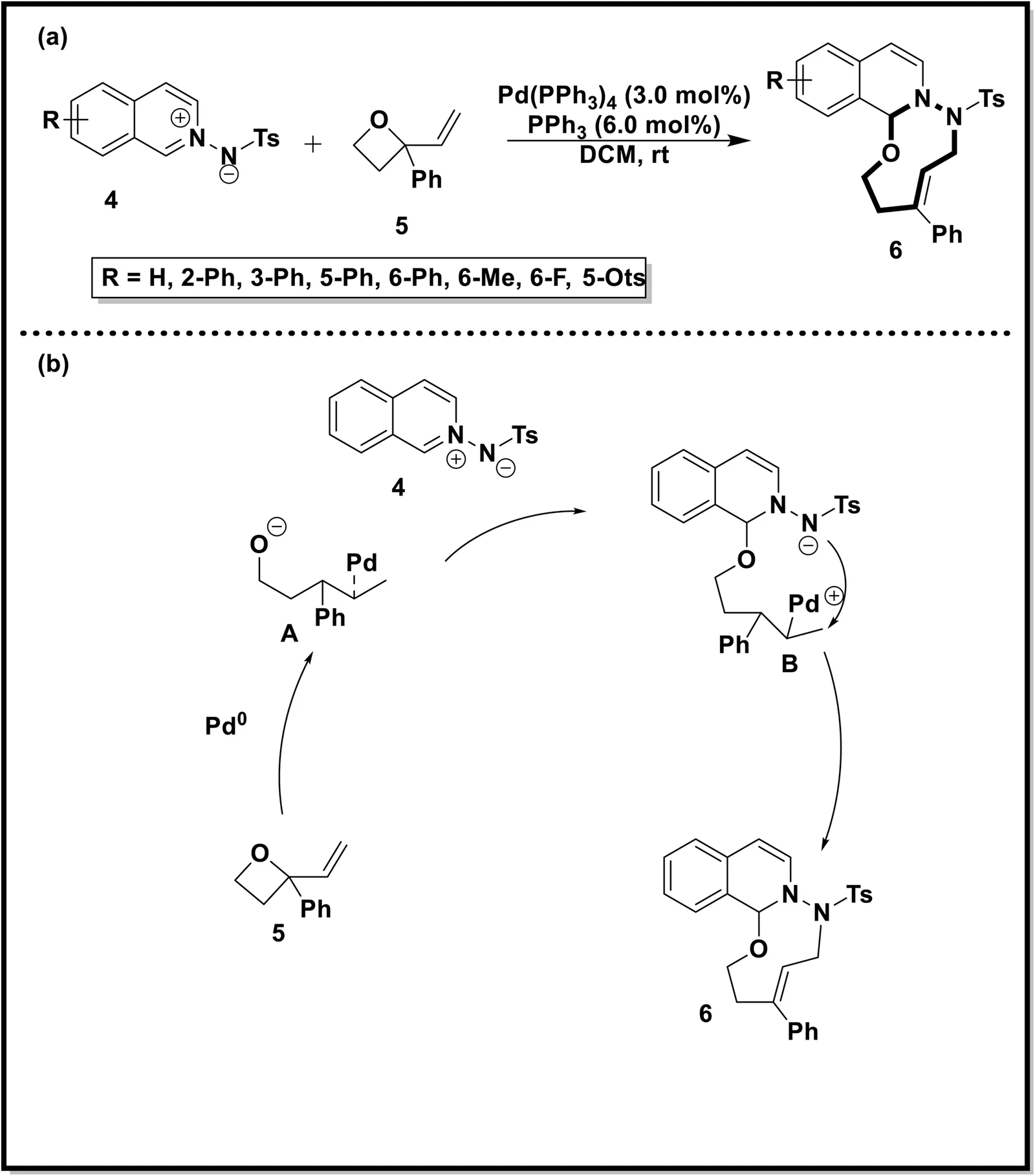
(a) A Pd-catalyzed synthesis of N,N,O-heterocycles; and (b) mechanistic investigation of Pd-catalyzed synthesis of nine-membered *N*,*N*,*O*-heterocycles *via* [6 + 3] CR.^21^

Xie *et al.* have explored a palladium-catalysed [6 + 3] dipolar cycloadditions of *N*-iminoisoquinolinium ylides 4 with 2-vinyl oxetanes 5 to afford nine-membered N,N,O-heterocycles 6 (Fig. 4a). Screening of palladium catalysts has identified Pd(PPh_3_)_4_ as the most efficient catalyst with an additional amount of triphenylphosphine (PPh_3_) as ligand further increased the yield of the cycloadducts 6. Solvent evaluation revealed that dichloromethane was the optimal medium. Increasing the amount of oxetane in [6 + 3] dipolar cycloadditions suppressed β-H elimination and furnished the desired product in 98% yield. Under these optimized conditions, various ylides and oxetanes delivered the cycloadducts in 53–98% yields with excellent diastereoselectivity.^21^

The mechanistic pathway of the Pd-catalyzed [6 + 3] dipolar cycloaddition demonstrated that the reaction is initiated by oxidative addition of Pd(0) to 5, generating the zwitterionic 1,6-dipolar intermediate A. Subsequently, the oxygen anion of A undergoes nucleophilic attack at the C1-position of *N*-iminoisoquinolinium ylide 4 to furnish the π-allyl palladium intermediate B. Finally, regioselective intramolecular amination at the terminal carbon of the π-allyl Pd moiety delivers the nine-membered N,N,O-heterocycle 6 along with regeneration of the Pd catalyst (Fig. 4b).

**Fig. 5:**
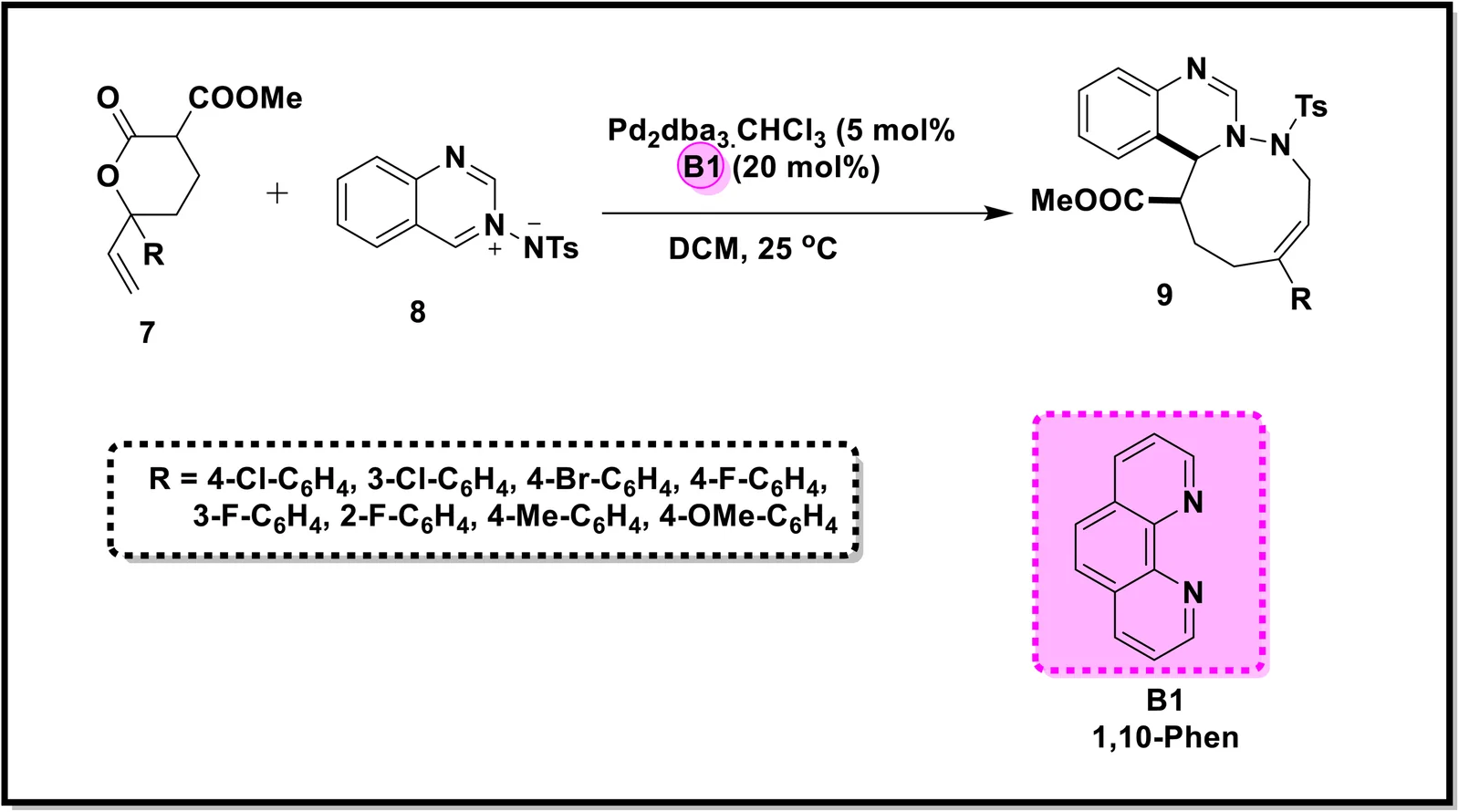
The synthesis of 1,2-dinitrogen-containing heterocycles by using [6 + 3] CR.^23^

Li *et al.* performed a Pd-catalyzed formal [6 + 3] cycloaddition of newly designed δ-vinylvalerolactones 7 with azomethine imines 8 to afford nine-membered 1,2-dinitrogen-containing heterocycles 9 (Fig. 5). Screening of ligands (dppe, PPh_3_, dppbz, Dpephos, Xantphos) revealed that 1,10-phenanthroline was the most efficient ligand, providing the cycloadduct in 98% yield with excellent diastereoselectivity (>20 : 1 d.r). A solvent evaluation determined that dichloromethane was the best solvent compared with CHCl_3_, DCE, toluene, THF, and MeCN. The study also confirmed that the Pd_2_(dba)_3_·CHCl_3_/B1 system was essential for reactivity and lowering the catalyst loading still maintained high yields. Under the optimized conditions, a broad range of δ-vinylvalerolactones and azomethine imines participated smoothly in the reaction, delivering the desired nine-membered heterocycles 9.^23^

**Fig. 6:**
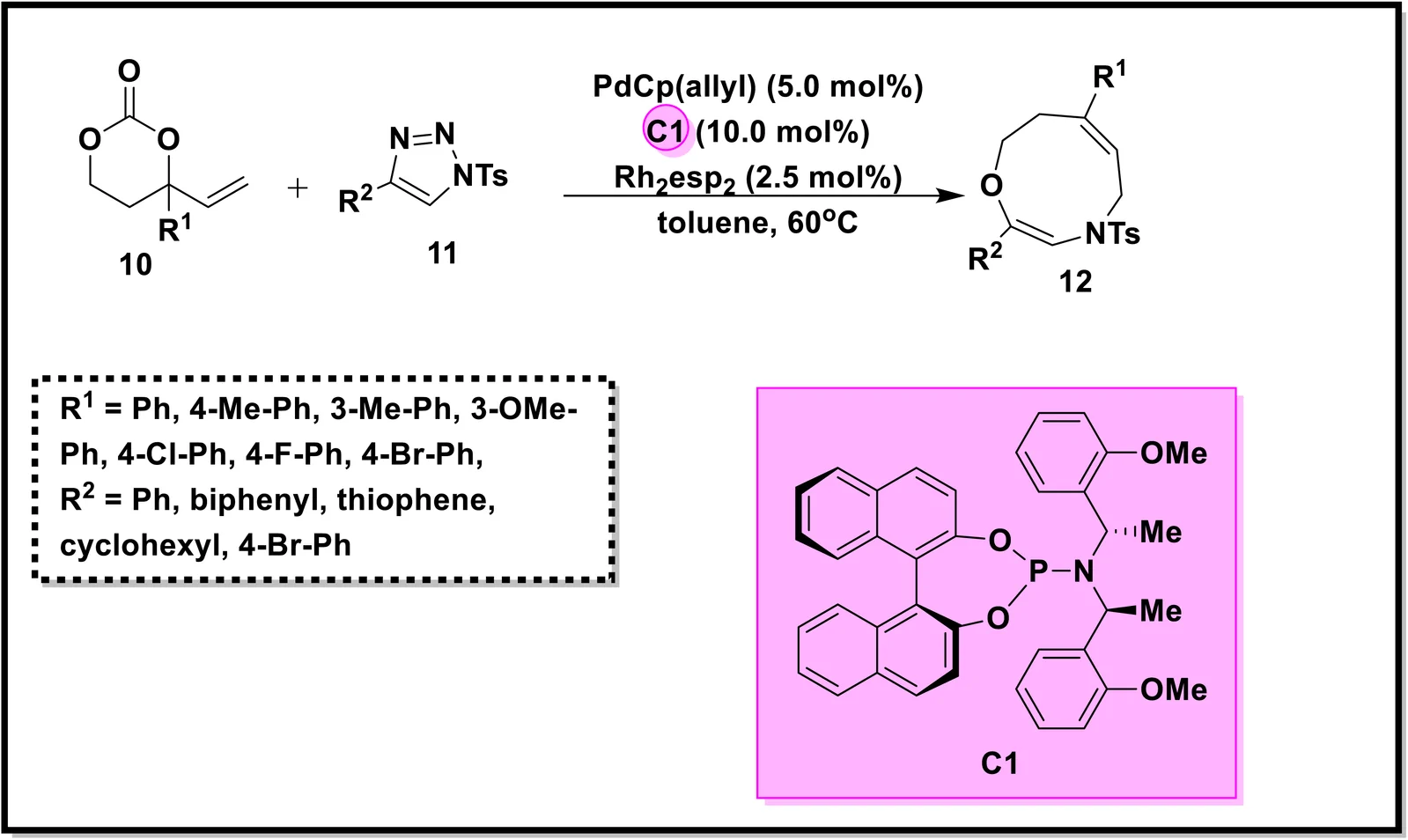
Synergistic Pd(0)/Rh(ii) catalyzed [6 + 3] CR for synthesis of monocyclic nine-membered N,O-heterocycles.^24^

Lee *et al.* reported the synergistic [6 + 3] dipolar cycloaddition reactions using two different metal catalyst Pd(0)/Rh(ii), between vinylpropylene carbonates 10 and *N*-sulfonyl-1,2,3-triazoles 11 enabling efficient access to monocyclic nine-membered heterocycles 12 (Fig. 6). Systematic evaluation of the ligand revealed that C1 in along with PdCp(allyl) and Rh_2_esp_2_ proved to be the optimal catalytic system. A diverse range of solvents was screened, and toluene was found to be the best solvent for this cycloaddition, whereas optimization of the catalyst loading ratio resulted in enhanced reaction efficiency. The study revealed that the cycloadditions proceeded effectively only with the combination of Pd and Rh as catalysts, indicating that single-metal catalysis (by Pd or Rh alone) was inadequate. The reactions provided a reliable method for building medium-sized heterocycles *via* [6 + 3] cycloadditions, achieving up to an 80% isolated yield of the target oxazonine products under optimal conditions.^24^

**Fig. 7:**
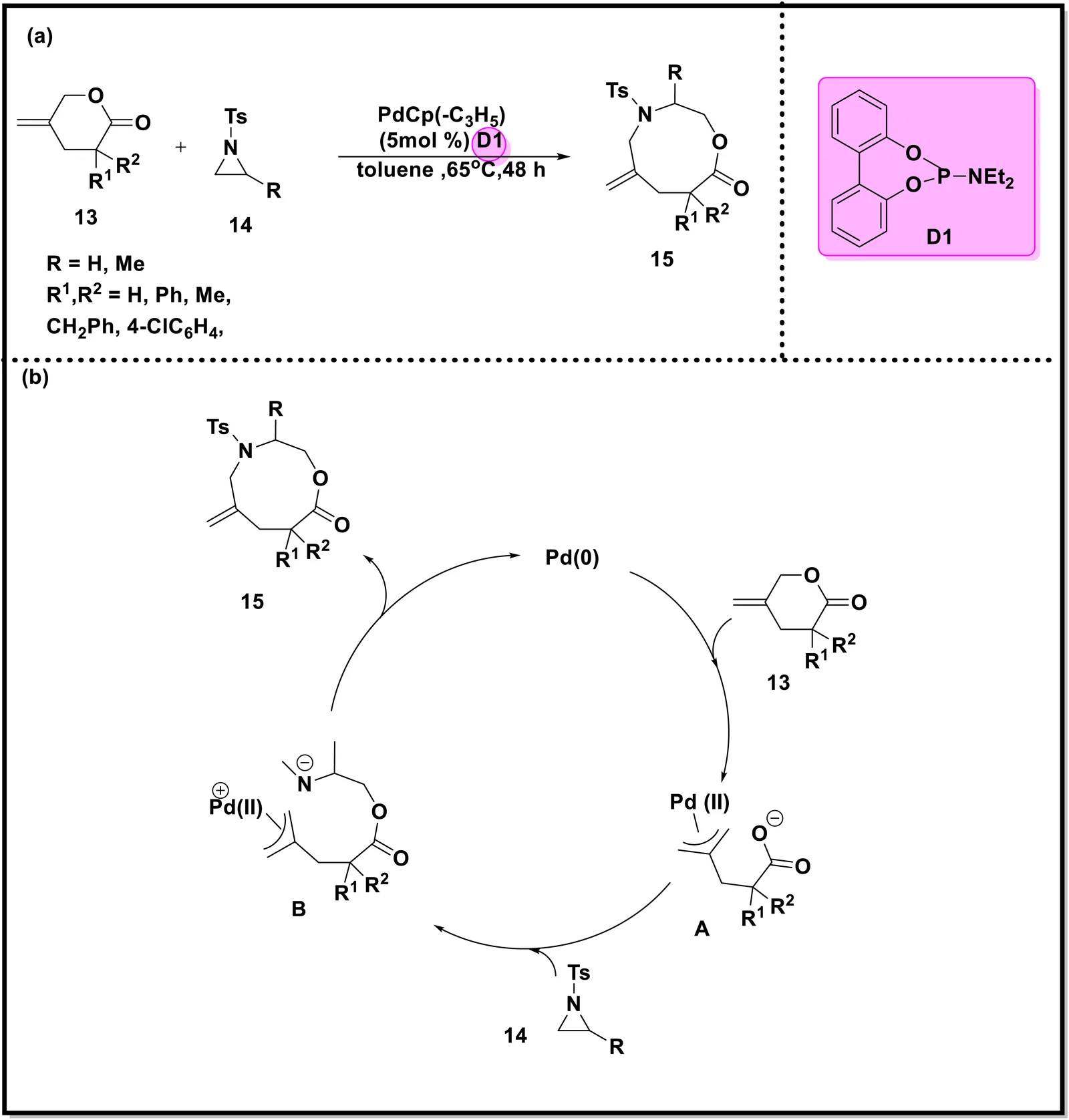
(a) [6 + 3] CR promoted the synthesis of 1,4-oxazonan-9-ones with a Pd catalyst (b) the mechanistic pathway of Pd-catalyzed synthesis of nine-membered 1,4-oxazonan-9-ones.^25^

Shintani *et al.*^25^ developed a Pd-catalyzed formal [6 + 3] ring-expansion of γ-methylidene-δ-valerolactones 13 with *N*-tosylaziridines 14 to access nine-membered 1,4-oxazonan-9-ones (azlactones) 15 (Fig. 7a). Ligand screening identified *N*,*N*-diisopropyl phosphoramidite (ligand D1) as the optimal ligand, outperforming PPh_3_, PCy_3_, BINAP, and dppf, while toluene at 80 °C afforded the best results with 86% yield. Under the optimized conditions, various γ-methylidene-δ-valerolactones and substituted aziridines were well tolerated, delivering the desired nine-membered azlactones in 53.87% yields with high regioselectivity.

The mechanistic pathway of the reaction revealed that the γ-methylidene-δ-valerolactone, upon oxidative addition with the Pd(0) catalyst, generated a π-allylpalladium carboxylate intermediate A. Subsequently, the carboxylate group of intermediate A regioselectively attacked the less hindered carbon of the *N*-tosylaziridine, affording intermediate B. The nitrogen atom of B then underwent an intramolecular allylic substitution by attacking the π-allylpalladium moiety, leading to the formation of the nine-membered 1,4-oxazonan-9-one ring with high regioselectivity. Finally, reductive elimination regenerated the active Pd(0) catalyst, affording the formal [6 + 3] ring-expansion product (Fig. 7b).^25^

**Fig. 8:**
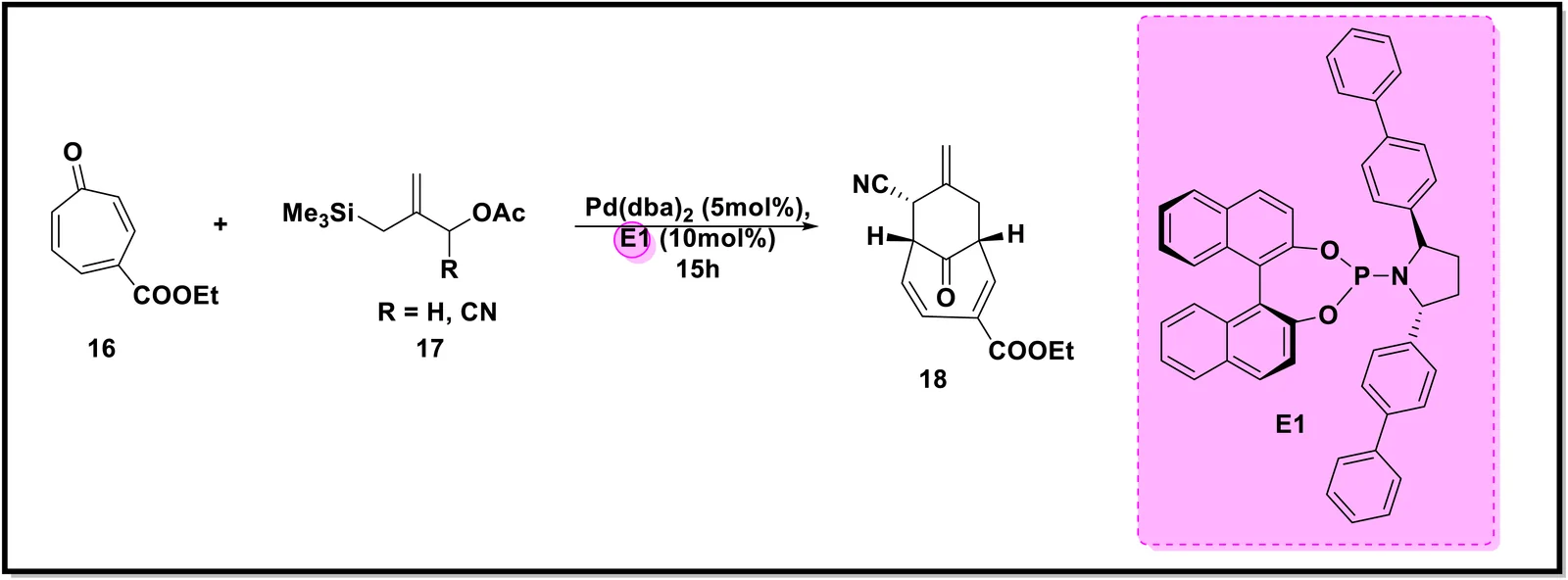
Synthesis of bicyclo[4.3.1]decadienes derivatives *via* [6 + 3] CR.^26^

Trost and his team developed enantioselective Pd-catalyzed formal [6 + 3] TMM cycloaddition of tropones 16 with cyano-substituted TMM 17 for the synthesis of bicyclo[4.3.1]decadienes 18. Screening of phosphoramidite ligands revealed that the cyclic pyrrolidine phosphoramidite ligand (E1) was the optimal ligand, affording the cycloadduct in 75% yield with near-perfect enantioselectivity (99% ee), whereas commercially available phosphoramidite ligands showed poor asymmetric induction. The optimized reaction employed Pd(dba)_2_ (5 mol%) and ligand (10 mol%) in toluene at 0–4 °C for 15 hours. Under these conditions, a wide range of electron-deficient and substituted tropones underwent exclusive [6 + 3] cycloaddition, furnishing bicyclo[4.3.1]decadienes with good to excellent yields and excellent regio-, diastereo-, and enantioselectivities. Furthermore, the resulting cycloadducts were readily converted into bicyclo[3.3.2]decadienes through a stereospecific thermal Cope rearrangement, with almost complete retention of enantiomeric purity, thereby expanding the synthetic utility of the methodology (Fig. 8).^26^

**Fig. 9:**
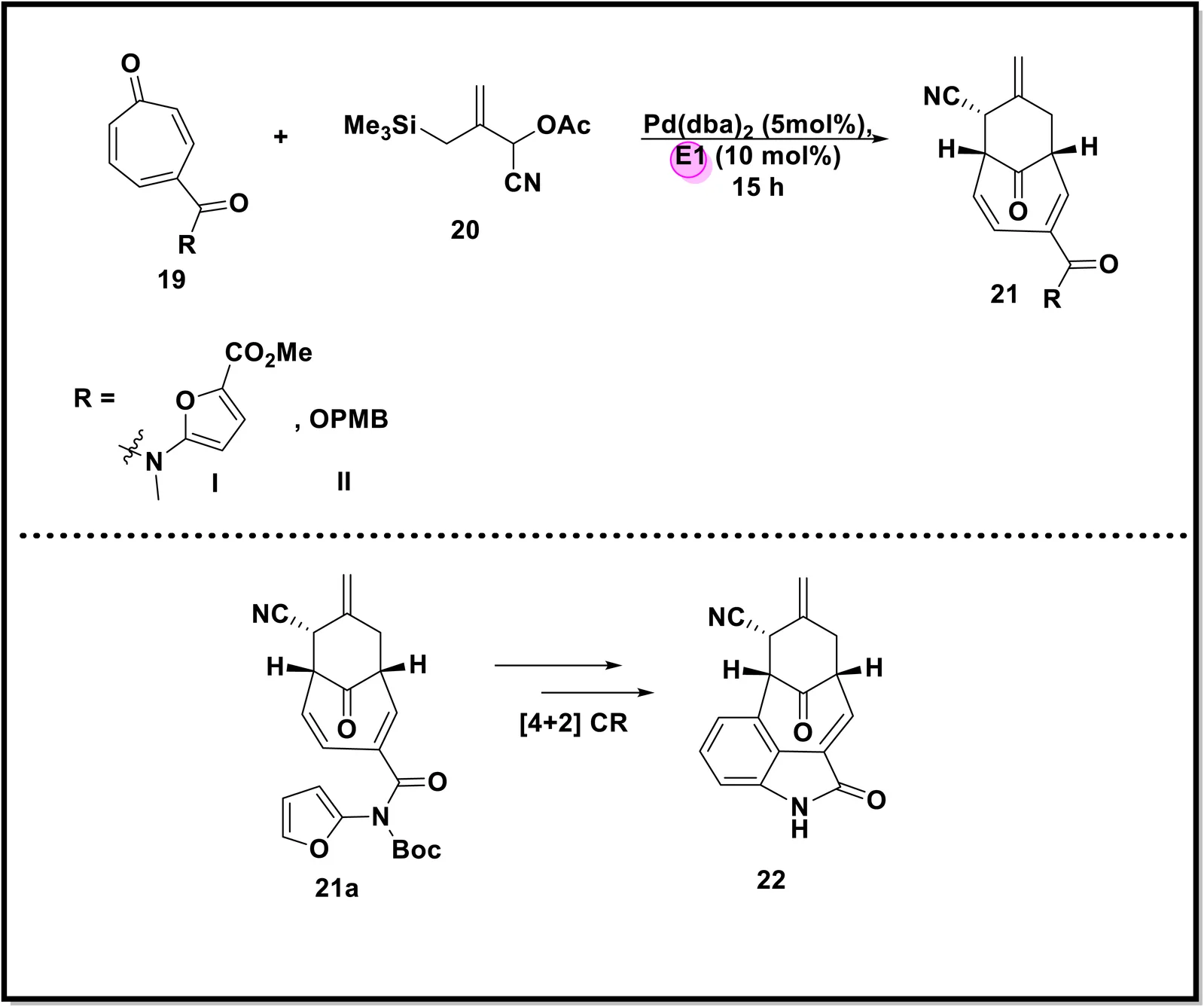
The role of Pd-catalyst in the synthesis of bicyclo[4.3.1]decadiene.^27^

Furthermore, his team developed a framework for Pd catalyst enantioselective [6 + 3] cycloaddition reaction between tropones 19 and TMM 20 to form bicyclo[4.3.1]decadiene 21 (Fig. 9). Tropone 19I afforded the cycloadduct in 60% yield with 94% ee, while tropone 19II gave the cycloadduct in 80% yield with similarly high enantioselectivity (94% ee). In both cases, the reaction proceeded with excellent regio- and diastereoselectivity, delivering a single cycloadduct. Further, by performing [4 + 2] CR author formed welwitindolinone 22 from 21a.^27^

Collectively, these studies demonstrate that palladium catalysts constitute one of the most versatile platforms for [6 + 3] cycloaddition reactions. Their success originates from the ability of Pd(0)/Pd(ii) species to undergo reversible oxidative addition/reductive elimination and to stabilize π-allyl or zwitterionic intermediates, thereby facilitating efficient ring construction. In most reported examples, phosphine- and phosphoramidite-based ligands play a decisive role in controlling both catalyst reactivity and stereoselectivity by creating a well-defined chiral environment around the palladium centre. Although Pd-catalyzed methodologies exhibit broad substrate scope and outstanding regio-, diastereo-, and enantioselectivity, their dependence on expensive phosphine ligands and preactivated substrates remains a limitation for broader synthetic applications.

#### 2.1.2. Copper-catalyzed [6 + 3] cycloaddition and formal [6 + 3] annulation reactions

**Fig. 10:**
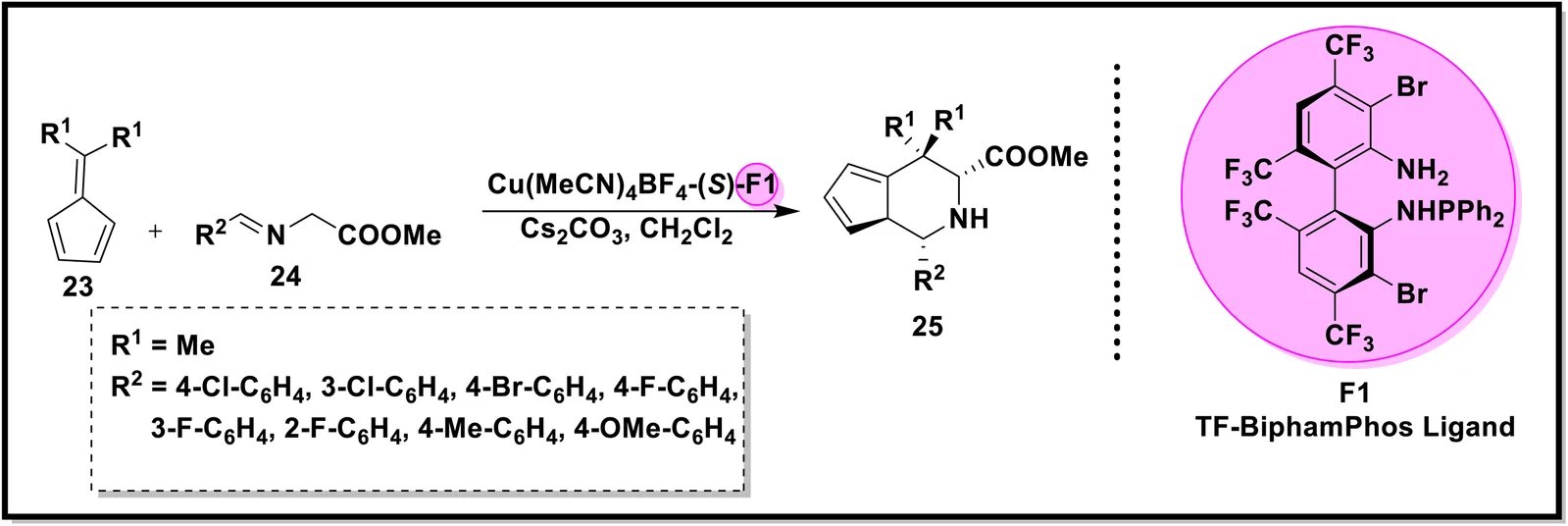
The Cu-catalyst efficiently promoted the synthesis of piperidine derivatives.^28^

Among transition metals, copper(i) complexes have proven highly effective in catalyzing [6 + 3] cycloaddition reactions due to their excellent ability to coordinate with π-systems, stabilize reactive zwitterionic intermediates, and promote the controlled generation and activation of dipolar species. Tao and Wang have developed a copper(i)-catalyzed asymmetric [6 + 3] cycloaddition of azomethine ylides 23 with fulvenes 24 to access enantioenriched piperidines 25. The [6 + 3] cycloadditions between symmetric/asymmetric fulvenes 24 and *N*-(4-chlorobenzylidene)glycine methyl esters 23a proceeded smoothly in the presence of Cu(MeCN)_4_BF_4_ and TF-BiphamPhos ligand F1, affording the functionalized piperidine in good yield with high diastereo- and enantioselectivity. Among the ligands screened, F1 bearing two bromine atoms at the 3,3′-positions of the biphenyl backbone afforded the best results (Fig. 10). Dichloromethane was identified as the optimal solvent, while Cs_2_CO_3_ proved to be the most efficient base furnished the product in up to 77% yield with 94% ee. The methodology tolerated a wide range of aromatic and aliphatic imino esters 24 as well as symmetric and unsymmetric fulvenes 22, affording cycloadducts 25 in 65–92% yields with excellent enantioselectivity (up to 99% ee). This Cu(i)/(*S*)-F1-catalyzed [6 + 3] cycloaddition thus provides an efficient route to highly substituted, enantioenriched piperidines 25.^28^

**Fig. 11:**
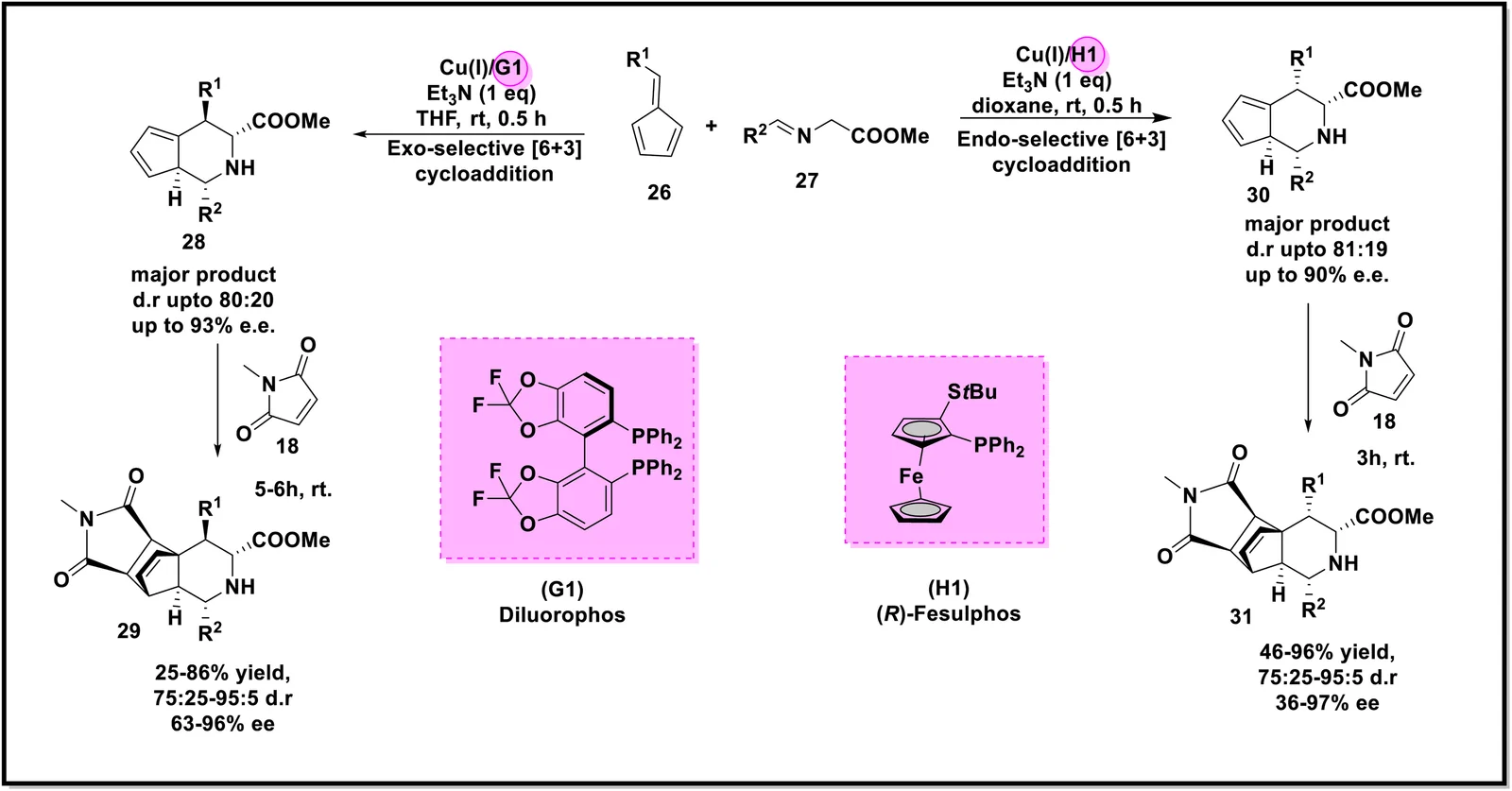
Cu catalyzed [6 + 3] cycloaddition reaction.^29^

Narayan *et al.* developed a series of catalytic enantioselective 1,3-dipolar cycloadditions of fulvene 26 using azomethine ylides 27 to construct ligand-based *exo*/*endo* cycloadducts *via* tandem [6 + 3]/[4 + 2] cycloaddition reaction (Fig. 11). The biphenylphos ligand (G1) offered *exo*-product with good yield up to 86% and high enantioselectivity up to 96%. While fesulphos ligand (H1) provided *endo*-product up to 96% yield with 97% ee.^29^

**Fig. 12:**
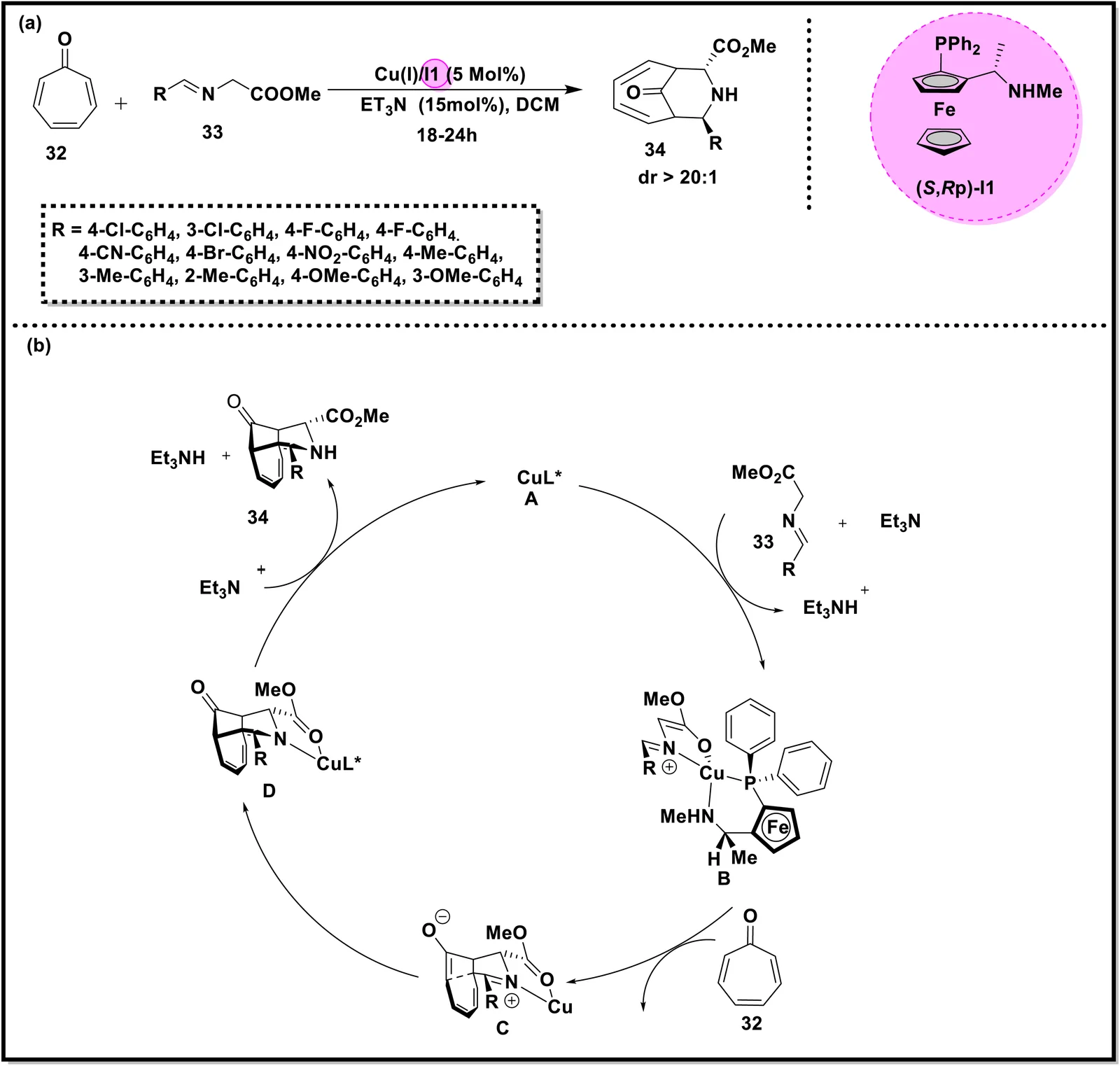
(a) Synthesis of bridged azabicyclo[4.3.1] decadiene derivatives *via* [6 + 3] CR; (b) the reaction pathway of Cu-catalyzed [6 + 3] CR.^30^

Teng *et al.* reported Cu(i)-catalyzed asymmetric [6 + 3] cycloadditions between tropone 32 and azomethine ylides 33 and to synthesize bridged azabicyclo[4.3.1] decadiene derivatives 34 (Fig. 12a). The reaction of tropone 32 with the glycine-derived Schiff base 33 was efficiently catalysed by a Cu(CH_3_CN)_4_BF_4_/PPh_3_ and Et_3_N in CH_2_Cl_2_ to furnish the [6 + 3] cycloadducts with high regio- and diastereoselectivity. The most efficient ligand in the study was the ferrocenyl-containing (*S*, *R*p)-I1, which produced the [6 + 3] cycloadduct in 76% yield along with >20 : 1 d.r. and 97% ee at −10 °C. Dichloromethane was the optimal solvent, and triethylamine served as the best base for aliphatic imino esters 33. Both aryl and alkyl imino esters 33 showed good compatibility, providing the desired products in 62–90% yields and up to 99% ee.^30^

The mechanistic pathway of the reaction revealed that the azomethine ylide, generated *in situ* in coordination with the Cu(i) catalyst, afforded tetracoordinated intermediate B, which underwent nucleophilic addition to the α-position of tropone 32 to form zwitterionic intermediate C. The steric environment created by the chiral ligand blocked one face of the ylide, directing the approach of tropone from the less hindered face and thereby controlling the stereoselectivity of the cycloaddition. Subsequently, intramolecular Mannich-type cyclization through a chair-like transition state furnished bridged bicyclic intermediate D with high regio-, diastereo-, and enantioselectivity. Finally, protonation of intermediate D afforded the azabicyclo[4.3.1]decadiene [6 + 3] cycloadduct 34 along with regeneration of the active Cu(i) catalyst (Fig. 12b).

**Fig. 13:**
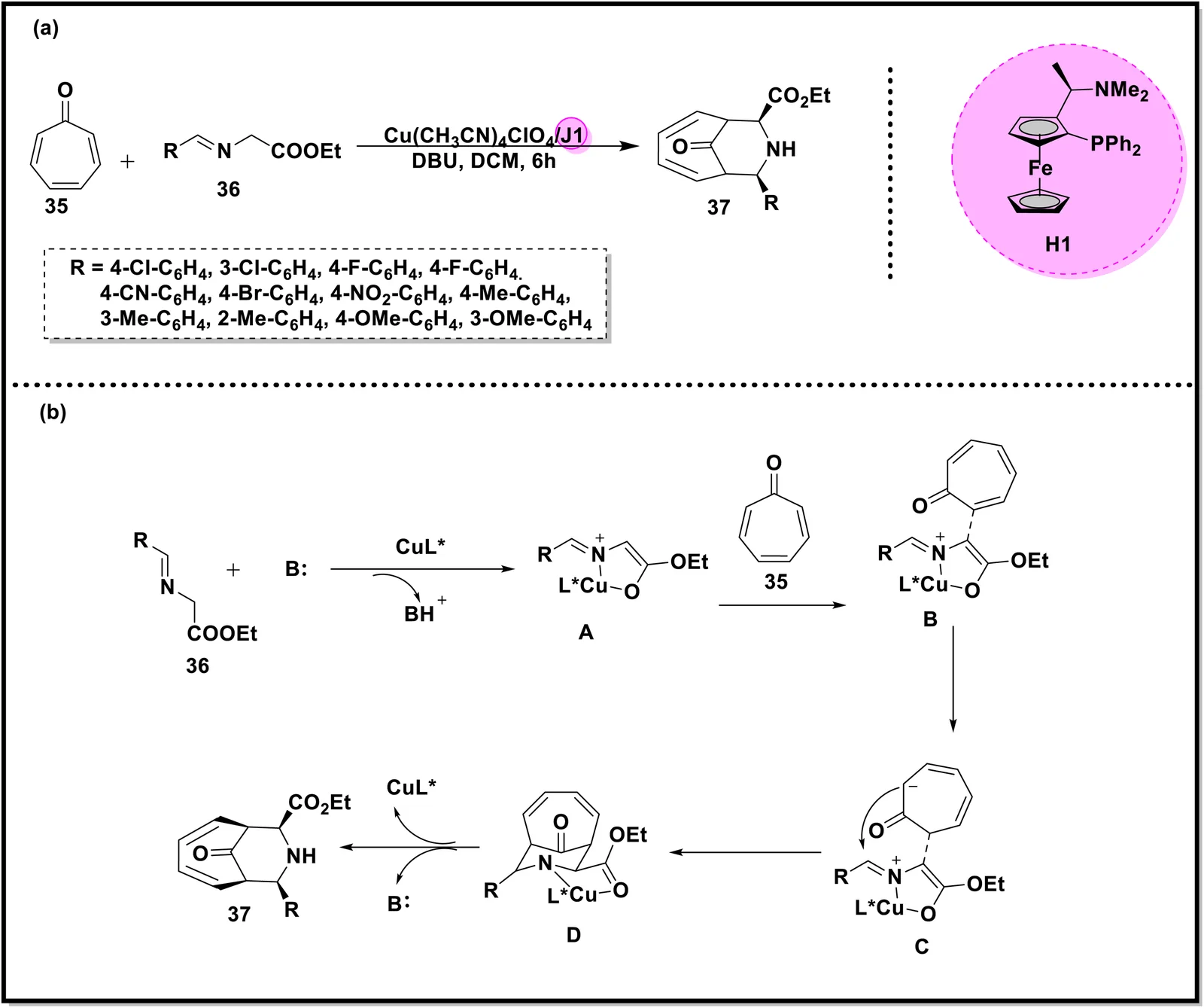
(a) Synthesis of piperidine-fused bicyclic heterocycles using AgOAc/PPh_3_ catalysed [6 + 3] cycloaddition; (b) the mechanistic pathway of Cu-catalyzed synthesis of piperidine-fused bicyclic heterocycles.^31^

Liu *et al.* performed the metal-catalyzed [6 + 3] cycloadditions between tropones 35 and azomethines 36 to synthesize piperidine-fused bicyclic heterocycles 37 (Fig. 13a). The reactions were well executed by exploring AgOAc/PPh_3_ as a catalyst at low temperature (−20 °C) in methanol and afforded [6 + 3] cycloadducts in good yields (up to 91%). The [6 + 3] cycloadditions proved effective for several α-imino esters with aryl substituents, yielding the desired 8-azabicyclo[4.3.1]deca-2,4-dienes with good diastereoselectivity (58% to 91%). The asymmetric variant of the [6 + 3] cycloaddition was tested using Cu(CH_3_CN)_4_ClO_4_/J1 as a catalyst, and yielded selective chiral products with high diastereo- and enantioselectivity (up to >20 : 1 d.r., 96% ee) with product yield ranging from 63% to 87%.^31^

The mechanistic pathway of the [6 + 3] cycloadditions revealed that in the presence of base and Cu(i)/ligand complex, the α-iminoester precursor generates a metallo-azomethine ylide intermediate A. Subsequently, nucleophilic addition of the metallated ylide to the electron-deficient tropone proceeds through transition state B to furnish zwitterionic intermediate C. The steric environment created by the chiral ligand directs the approach of tropone from the less hindered backside, thereby controlling the stereochemistry of the [6 + 3] cycloaddition. Thereafter, intramolecular nucleophilic attack of the carbanion onto the si-face of the imine moiety forms bicyclic intermediate D. Finally, protonation of intermediate D affords the piperidine-fused bicyclic [6 + 3] cycloadduct 37 along with simultaneous regeneration of the Cu(i) catalyst for the next catalytic cycle (Fig. 13b).

**Fig. 14:**
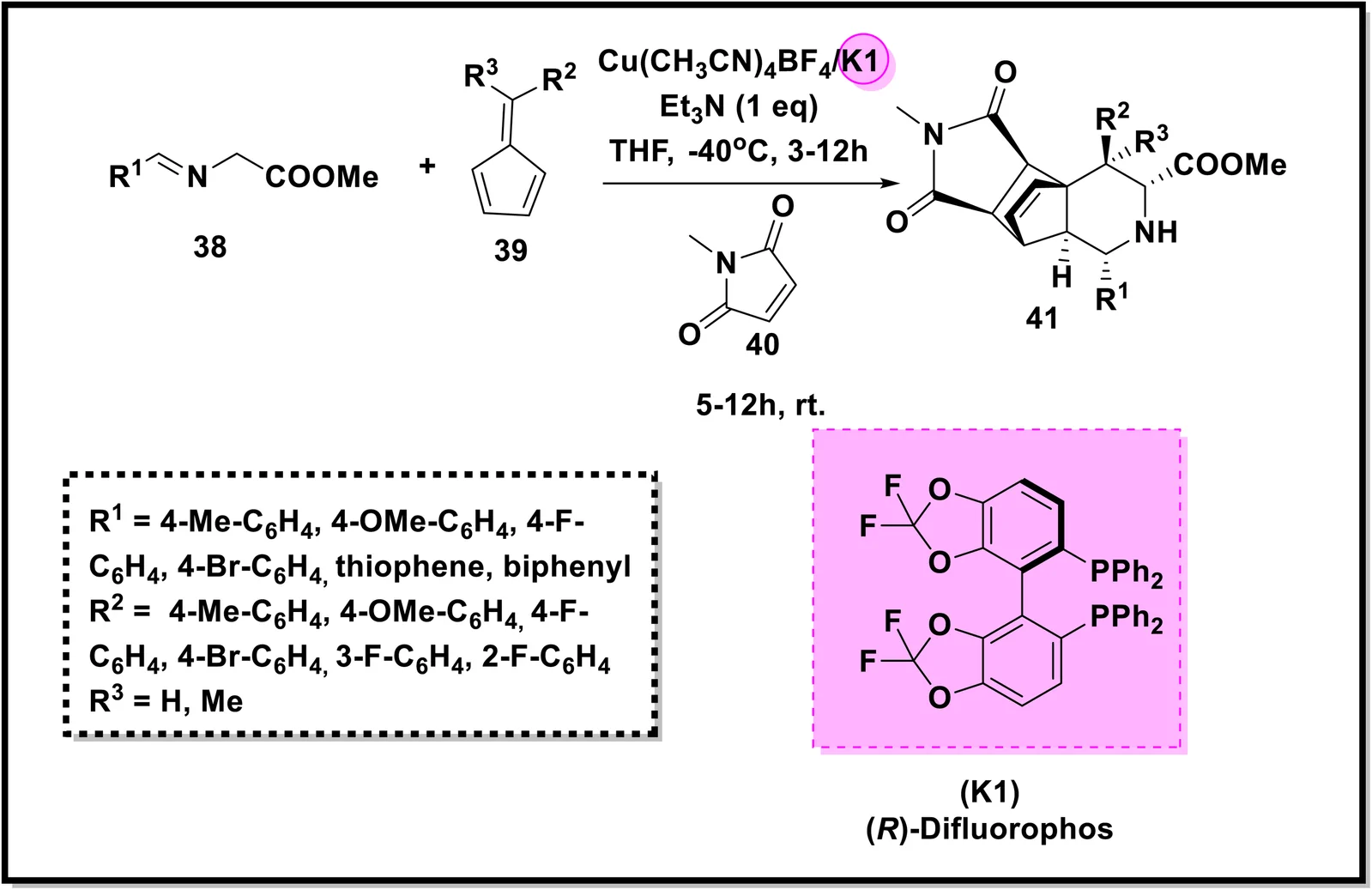
[6 + 3] CR promoted the synthesis of piperidine derivatives with a Cu catalyst.^32^

Potowski and coworkers treated azomethine ylides 38 with fulvenes 39 in the presence of a Cu-catalyst for the synthesis of highly enantio-enriched piperidine derivatives 41 (Fig. 14). The reaction of glycine ester imine 38 with fulvene 39 was systematically optimized by screening different Cu(i) salts along with a range of chiral ligands. Among the tested conditions, Cu(CH_3_CN)_4_BF_4_ combined with the (*R*)-difluorophos as ligand K1 delivered the optimal outcome, affording the *exo*-cycloadduct in 80% yield and 91% ee in methanol at 0 °C. Further studies identified methanol as the optimal solvent and Et_3_N as the most effective base. When the reaction was conducted at −40 °C in THF, both diastereo- and enantioselectivity increased, providing the [6 + 3] cycloadducts in 76% yield with 85 : 15 d.r. and 95% ee.^32^

**Fig. 15:**
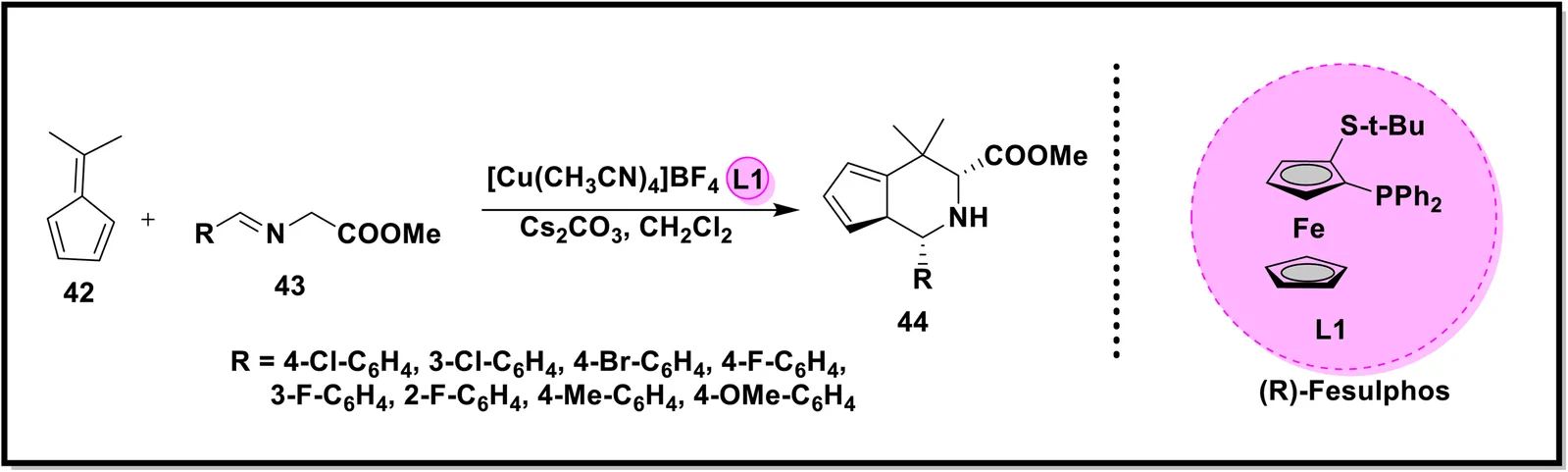
The reaction pathway of Cu-catalyzed [6 + 3] cycloadditions for synthesis of piperidine derivatives 44.^33^

Potowski and coworkers also explored the asymmetric [6 + 3] cycloaddition reactions of fulvenes 42 and azomethine ylides 43 for the synthesis of piperidine derivatives 44 (Fig. 15). The asymmetric [6 + 3] cycloaddition reactions of azomethine ylide 43 with fulvene 42 were optimized by exploring the various Cu(i) catalysts and chiral ligands. The excellent results were achieved using [Cu(CH_3_CN)_4_]BF_4_/(*R*)-L1/Et_3_N in 1,4-dioxane at room temperature, which yielded the [6 + 3] cycloadduct (80%) with high stereoselectivity (87 : 13 d.r., 92% ee). Various azomethine ylides 43 and fulvenes 42 have been explored in these [6 + 3] cycloadditions to yield functionalized piperidine derivatives 44 in good to excellent yields (62–96%) and high enantioselectivity (up to 97% ee).^33^

**Fig. 16:**
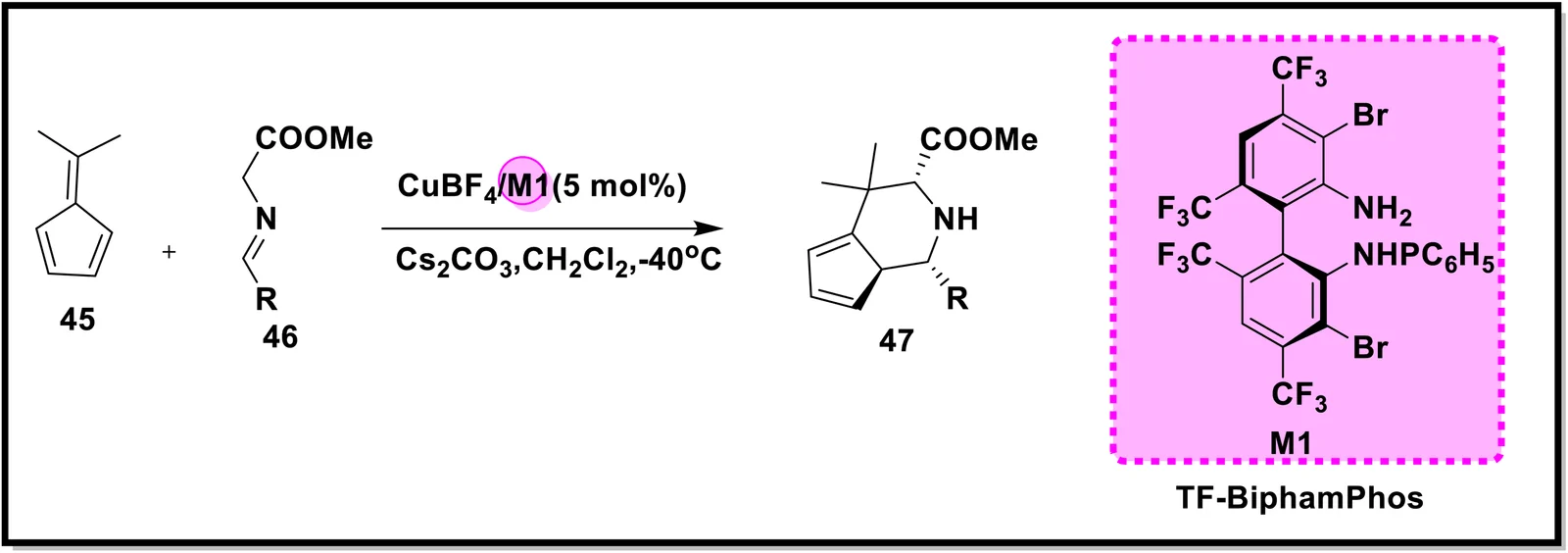
Synthesis of six-membered piperidine derivatives using [6 + 3] CR.^34^

He *et al.* performed asymmetric [6 + 3] cycloaddition of fulvenes 45 with imino esters 46 in the presence of a Cu catalyst to form highly diastereoselective and enantioselective piperidine derivatives 47 (Fig. 16). Out of the fifteen tested ligands, M1 bearing two bromine substituents at the 3,3′-position of the TF-BiphamPhos backbone was the most efficient ligand. A comprehensive evaluation of the solvents determined that dichloromethane as a best solvent in these [6 + 3] cycloadditions. The study revealed that Cs_2_CO_3_ was the ideal base and promoted the reaction without any detrimental effect on diastereoselectivity and enantioselectivity. The optimal condition targets the desired product yield up to 87% with high enantioselectivity (99% ee).^34^

Unlike palladium catalysts, Cu(i)-based systems predominantly operate through metallo-azomethine ylide intermediates rather than π-allyl complexes. The reviewed studies consistently demonstrate that the stereochemical outcome is primarily governed by the chiral ligand rather than the copper salt itself, with several catalytic systems delivering products in up to 99% ee. Copper catalysis therefore represents one of the most effective approaches for asymmetric [6 + 3] cycloaddition reactions. Nevertheless, the majority of reported examples remain largely confined to fulvenes and tropones, indicating that expanding the scope to other 6-atom synthons remains an important future challenge.

#### 2.1.3. Silver-catalyzed [6 + 3] cycloaddition and formal [6 + 3] annulation reactions

Silver(i) salts have emerged as effective catalysts in [6 + 3] cycloaddition reactions, offering a complementary alternative to copper-based systems. Owing to their mild Lewis acid character and strong affinity toward nitrogen-containing dipoles, silver catalysts efficiently activate azomethine ylides for cycloaddition with 6-atom π-partners such as tropone and fulvenes. These reactions proceed with high regio- and stereoselectivity, and when combined with chiral ligands, enable the asymmetric variants, providing rapid access to diverse fused heterocyclic frameworks of biological significance.

**Fig. 17:**
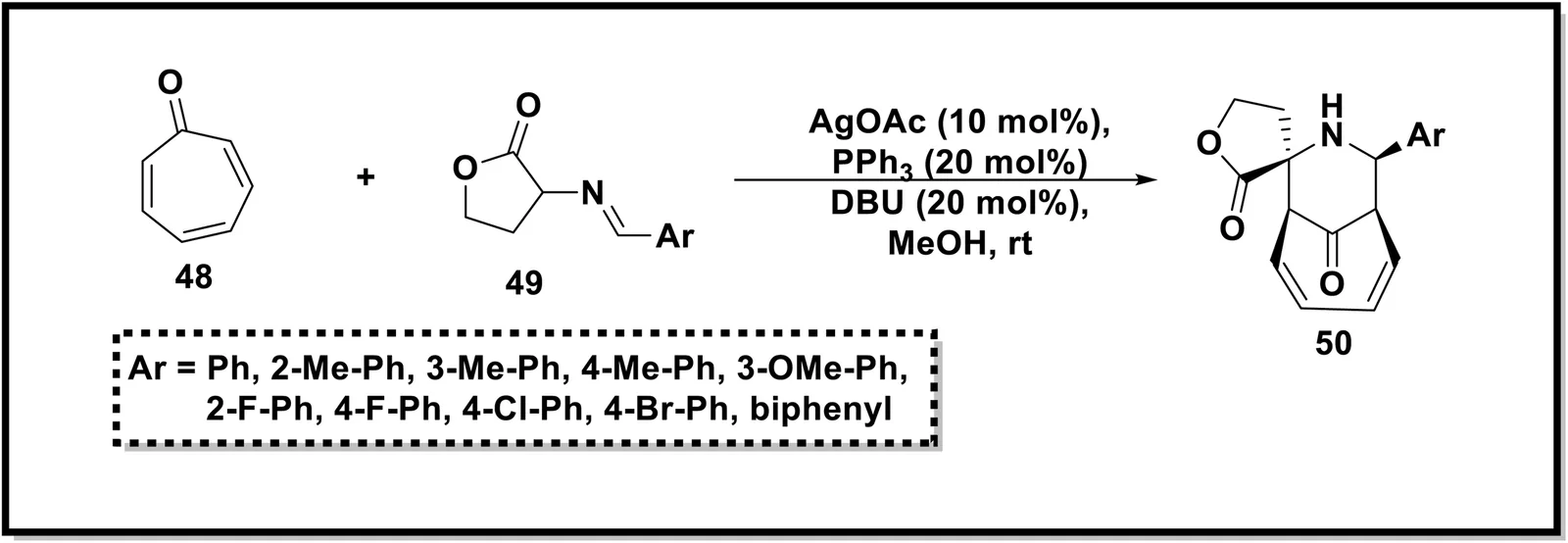
Ag catalyst synthesis of tricyclic spiropiperidine derivatives.^35^

Wu *et al.* have developed an Ag-catalyzed diastereoselective [6 + 3] cycloaddition of tropones 48 with homoserine lactone-derived azomethine ylides 49 to construct tricyclic spiropiperidine derivatives 50 (Fig. 17). Among the various silver salts examined, AgOAc in combination with PPh_3_ was identified as the most efficient catalytic system, providing the desired product in excellent yield with complete diastereocontrol. A systematic screening of bases revealed that DBU was the most suitable base, effectively promoting the cycloaddition without affecting the diastereoselectivity of the reaction. Solvent optimization studies showed that methanol was a superior solvent compared to other solvents such as dichloromethane, THF, and acetonitrile. Under the optimized conditions, the reaction delivered the corresponding tricyclic spiropiperidine up to 92% yield with excellent diastereoselectivity (d.r. > 20 : 1).^35^

**Fig. 18:**
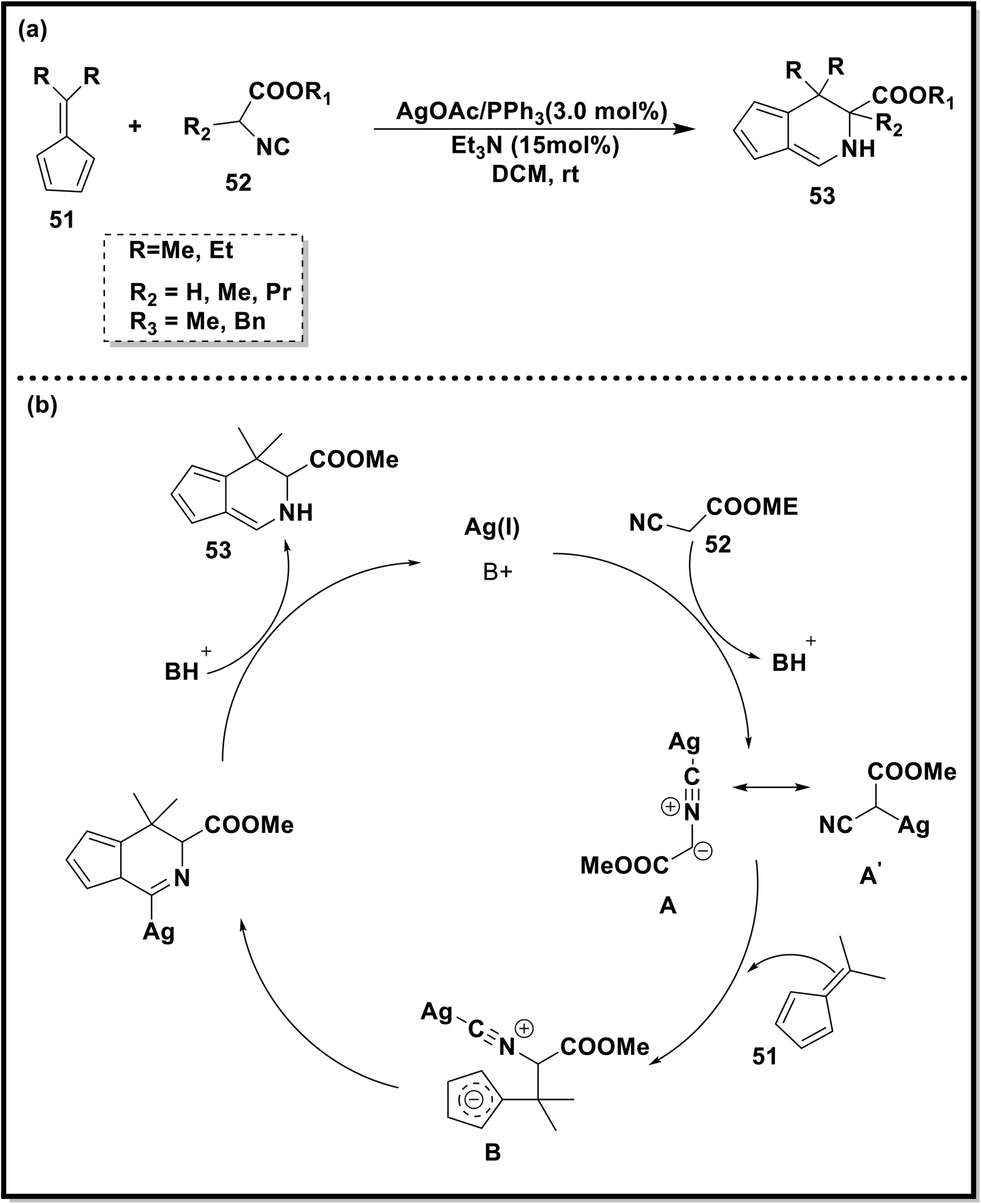
(a) Synthesis of fused dihydropyridine rings using [6 + 3] cycloadditions; (b) mechanistic investigation of Ag-catalyzed synthesis of fused dihydropyridines *via* [6 + 3] CR.^36^

He and Wang reported Ag(i)-catalyzed [6 + 3] cycloadditions between and fulvenes 51 and isocyanoacetates 52 for the synthesis of fused dihydropyridines 53 in good yields with exclusive regioselectivity (Fig. 18a). The most effective conditions determined through optimization involved the use of AgOAc/PPh_3_ as catalytic system in dichloromethane and Et_3_N as a mild base to afford [6 + 3] cycloadducts in good yields (95%). The [6 + 3] cycloadditions required both the base and the ligand, as control experiments indicated that Cu(i) salts alone could not promote the transformation. The reaction was compatible with diverse symmetrical and unsymmetrical fulvenes as well as sterically demanding isocyanoacetates, yielding the [6 + 3] cycloadducts in good yields. The asymmetric variants of [6 + 3] cycloadditions using chiral catalysts such as AgOAc-TF-BiphamPhos yielded the fused dihydropyridines 53 in good yields (71%) but with poor enantioselectivity (28% ee).^36^

The proposed mechanistic pathway is outlined in Fig. 18b. Initially, Ag(i)-catalyzed metalation and α-deprotonation of isocyanoacetate 52 generated nucleophilic intermediate A or its tautomer A′. Subsequently, intermediate A underwent nucleophilic attack on fulvene 51 to furnish the zwitterionic intermediate B. Further, intermediate B underwent intramolecular cyclization followed by protonation and isomerization to afford the fused dihydropyridines 53 along with regeneration of the Ag(i) catalyst.

**Fig. 19:**
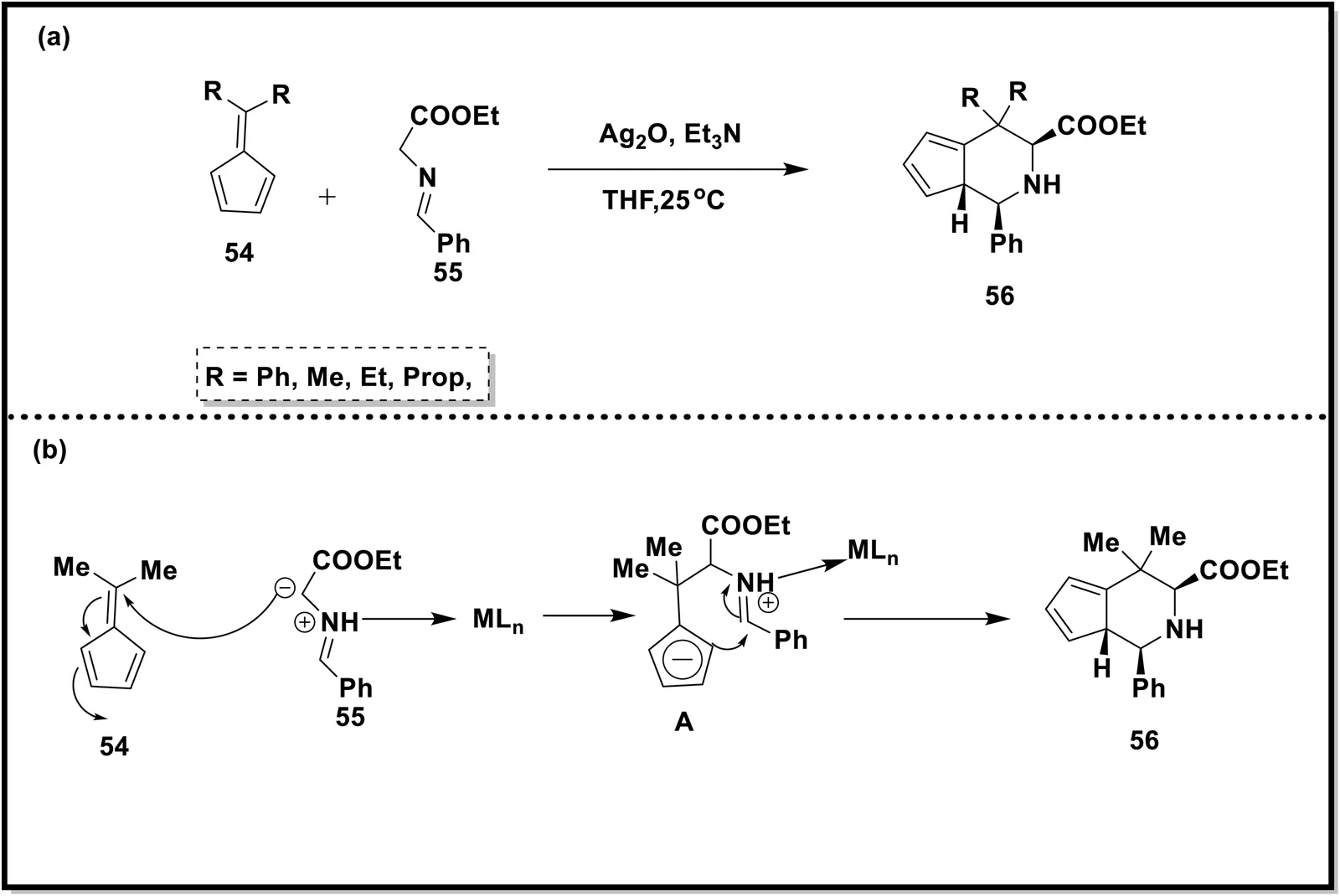
(a) The reaction pathway explored the role of silver metal in [6 + 3] CR; (b) the reaction pathway of Ag-catalyzed [6 + 3] CR.^37^

Hong *et al.* have explored the [6 + 3] cycloadditions of fulvenes 54 with *N*-alkylidene glycine esters 55, introducing an effective pathway to access functionalized tetrahydro-pyrindine-based heterocycles 56 (Fig. 19a). Such pyrindine-based heterocycles are present in various natural products such as delavayine and incarvillateine. The preliminary investigation revealed that the reaction of *N*-benzylidene glycine ethyl ester 55 with 6,6-dimethylfulvene (54) produced the functionalized pyrindine 56 in 80% yield and excellent stereoselectivity. The reactions were promoted by metalloazomethine ylides generated under different conditions, among which Ag_2_O/Et_3_N in THF proved to be the most effective catalyst, affording the [6 + 3] cycloadducts in good yields (up to 92%).^37^

A plausible mechanism for the hetero-[6 + 3] cycloadditions involved the initial deprotonation/metalation of *N*-benzylidene glycine ethyl esters 56 to generate metalloazomethine ylides 55. This is followed by the nucleophilic addition of the deprotonated *N*-benzylidene glycine ethyl esters 55 to the exocyclic C6 position of 6,6-dimethylfulvene 54 to form zwitterionic intermediate A. Subsequent intramolecular nucleophilic cyclization of the previously generated carbanionic centre on the iminium carbon furnished the bicyclic-pyrindine framework. The reactions proceeded through a chair-like transition state, leading to high stereoselectivity, and final proton transfer/demetalation afforded functionalized bicyclic-pyrindines in good yields 56 (Fig. 19b).

Silver catalysts primarily function as mild Lewis acids that facilitate the generation of metallo-azomethine ylides and activate electron-deficient π-systems under relatively mild conditions. Compared with copper catalysts, Ag(i)-based systems generally exhibit comparable regio- and diastereoselectivity but lower levels of enantioinduction in asymmetric transformations. Consequently, silver catalysts are particularly attractive for operationally simple and highly efficient racemic or diastereoselective [6 + 3] cycloaddition reactions, whereas further catalyst development is required to improve asymmetric variants.

#### 2.1.4. Chromium metal-based catalyzed [6 + 3] cycloaddition reactions

Chromium and its reagents have been efficiently explored in [6 + 3] cycloaddition reactions. Tricarbonylchromium (0) complexes are well known for activating both the six-atom and three-atom components through coordination, facilitating cycloaddition under photochemical or thermal conditions. The complexation of chromium with cycloheptatriene enhances its reactivity toward three-atom partners such as azirines and carbonyl ylides, enabling the construction of bicyclic and polycyclic ring systems with high selectivity.

**Fig. 20:**
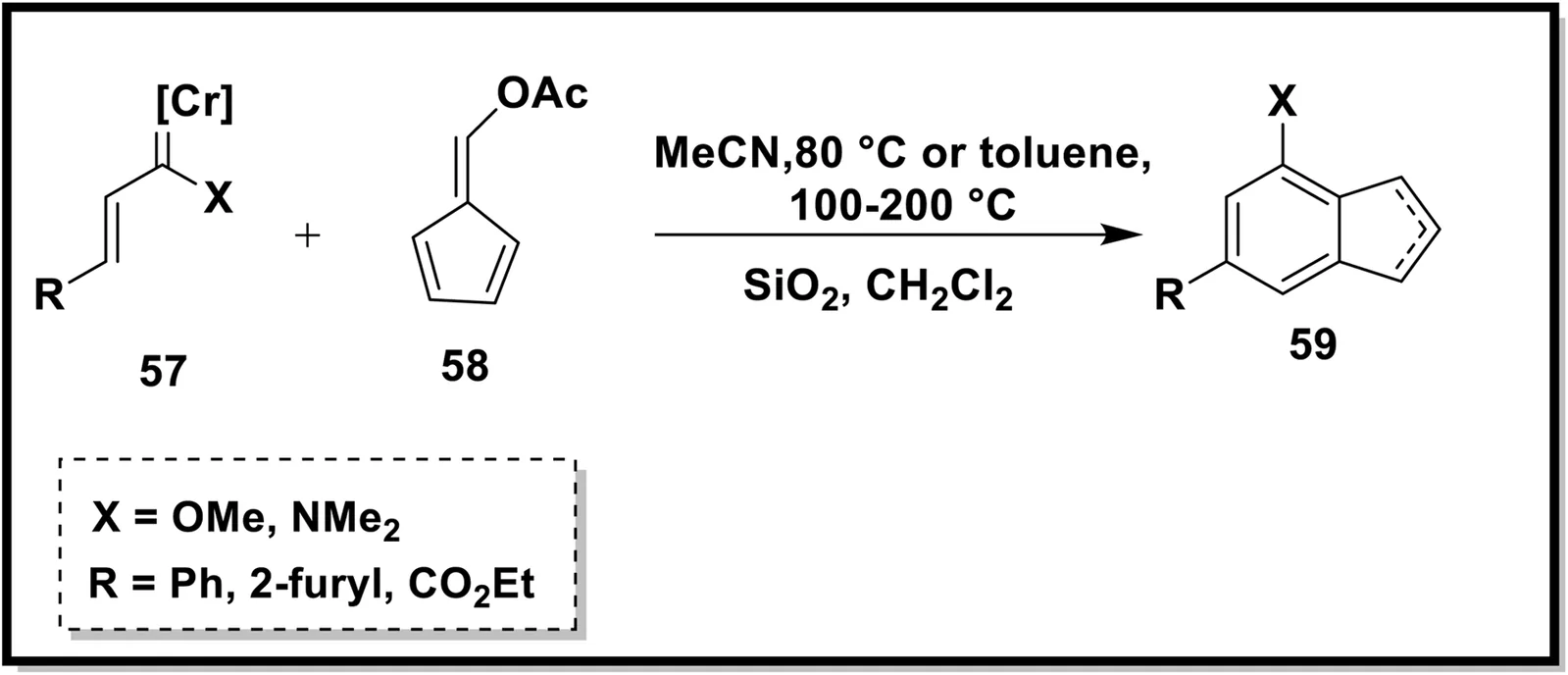
The synthesis of substituted indenes using [6 + 3] CR.^38^

Barluenga and his group reported [6 + 3] cycloaddition of Fischer carbene complexes 57 and fulvenes 58 to yield substituted indenes 59 (Fig. 20). The reaction involved the treatment of 6-acetoxyfulvene with chromium alkenyl carbene complexes under optimized conditions (MeCN, 80 °C or toluene, 100–120 °C), producing indene derivatives in 65–75% yield. The study revealed that this transformation proceeds *via* nucleophilic 1,2-addition of fulvenes to the carbene carbon, followed by a [1,2]-Cr(CO)_5_ shift, highlighting a new reactivity pathway for Fischer carbene complexes.^38^

In contrast to Pd-, Cu-, and Ag-catalyzed systems, Cr-mediated [6 + 3] cycloadditions primarily proceed through substrate activation *via* metal coordination. Although chromium complexes enable unique transformations of conjugated π-systems, their broader application is limited by the need for chromium carbonyl complexes and thermal or photochemical activation.

#### 2.1.5. Comparative analysis of metal-catalyzed [6 + 3] cycloaddition reactions

Although remarkable advances have been achieved in metal-catalyzed [6 + 3] cycloaddition chemistry, each catalytic platform exhibits distinct mechanistic characteristics that influence reaction efficiency and selectivity. Palladium catalysts generally operate through π-allyl intermediates generated by oxidative addition and are particularly effective for achieving high regio- and enantioselectivity through ligand-controlled transition states. In contrast, Cu(i)-based catalysts generate metallo-azomethine ylides and provide exceptional stereochemical control in asymmetric transformations when combined with suitable chiral phosphine ligands. Silver catalysts primarily function as mild Lewis acids, facilitating dipole generation and cycloaddition under relatively mild conditions but generally displaying lower enantioselectivity than copper systems. Chromium-mediated processes proceed through π-complex activation and offer complementary reactivity; however, their dependence on stoichiometric metal complexes and photochemical or thermal activation limits their synthetic practicality (Table 1). Overall, ligand design, substrate electronics, and catalyst activation mode are the principal factors governing chemo-, regio-, and stereoselectivity in metal-catalyzed [6 + 3] cycloaddition reactions. Future developments should focus on earth-abundant metals, sustainable catalytic systems, lower catalyst loadings, and broader substrate compatibility while maintaining high levels of stereocontrol.

**Table 1:** A brief analysis and comparison between metal catalysis for [6 + 3] cycloaddition reactions

Catalyst	Activation mode	Key intermediate	Advantages	Limitation	Selectivity characteristics
Pd	π-Coordination, oxidative addition/reductive elimination	π-Allyl Pd, zwitterionic intermediate	Broad substrate scope, excellent functional-group tolerance, high asymmetric induction	Expensive catalyst, phosphine ligands; often requires activated substrates	Excellent chemo-, regio-, diastereo-, and enantioselectivity
Cu(i)	Metallo-azomethine ylide generation	Cu-bound azomethine ylide	Good asymmetric catalysis, mild conditions, high ee	Limited substrate diversity (mainly fulvenes/tropones)	Excellent diastereo- and enantioselectivity
Ag(i)	Lewis acid activation/Metallo-azomethine ylide	Ag-bound ylide/zwitterionic intermediate	Mild conditions, simple operation, broad functional-group tolerance	Lower enantioselectivity than Cu catalysts	Excellent regio- and diastereoselectivity; moderate ee
Pd/Rh	Cooperative dual-metal activation	Pd π-allyl/Rh carbene intermediates	Access to otherwise inaccessible nine-membered rings	More complex optimization and higher catalyst cost	High chemo- and regioselectivity
Cr(CO)_3_	π-Complex activation	Cr-coordinated intermediate	Unique reactivity toward conjugated π-systems	Stoichiometric metal complexes; thermal/photochemical activation	Good regioselectivity; limited modern applicability

### 2.2. Non-metal catalyzed [6 + 3] cycloaddition reactions

Beyond transition metal catalysis, non-metal-catalyzed [6 + 3] cycloaddition reactions have emerged as sustainable and cost-effective alternatives. Organocatalysts such as Brønsted acids, Lewis acids, and *N*-heterocyclic carbenes, along with photochemical and thermal conditions, have been successfully employed to activate the reaction components, offering advantages of lower toxicity and environmental compatibility.

#### 2.2.1. Organocatalyst-catalyzed [6 + 3] cycloaddition and formal [6 + 3] annulation reactions

**Fig. 21:**
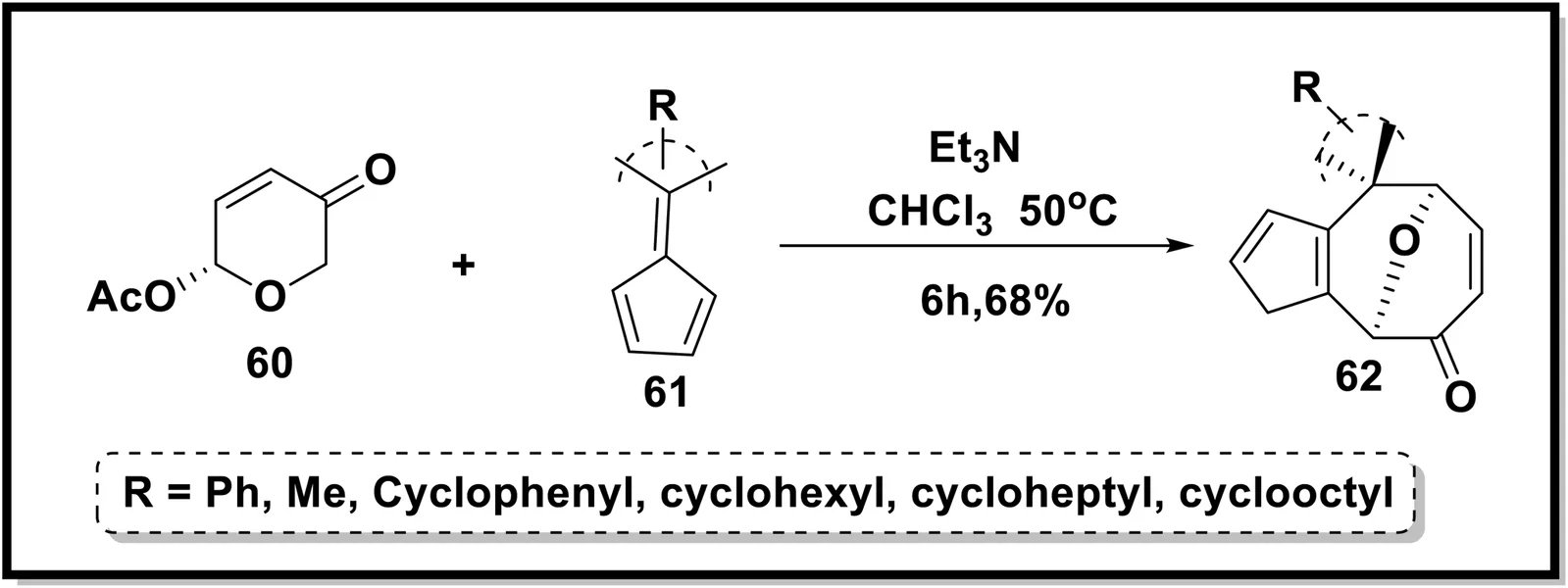
The organocatalysed mediated [6 + 3] CR for synthesis of oxabridged polycyclic scaffold.^39^

Kuthanapillil and his team^12^ disclosed the base-catalyzed [6 + 3] cycloadditions of pentafulvenes 60 with 3-oxidopyrylium betaine 61, enabling efficient access to oxabridged polycyclic frameworks 62 (Fig. 21). The systematic evaluation revealed that the [6 + 3] reaction carried out in chloroform with a base as a catalyst afforded [6 + 3] adducts exclusively and in moderate to good yields. Different types of solvents were tested in these [6 + 3] cycloadditions, and non-polar solvents such as chloroform were observed as the most effective solvent in these [6 + 3] cycloadditions, whereas polar solvents resulted in a loss of selectivity for the [6 + 3] cycloadduct. Additionally, fulvenes substituted with diphenyl, dimethyl, or cycloalkylidene groups reacted efficiently and produced the oxabridged [6 + 3] cycloadducts. The best results were achieved using triethylamine as a catalyst in chloroform at room temperature to yield the [6 + 3] cycloadducts exclusively (no side adducts) in good yield (83%).^39^

**Fig. 22:**
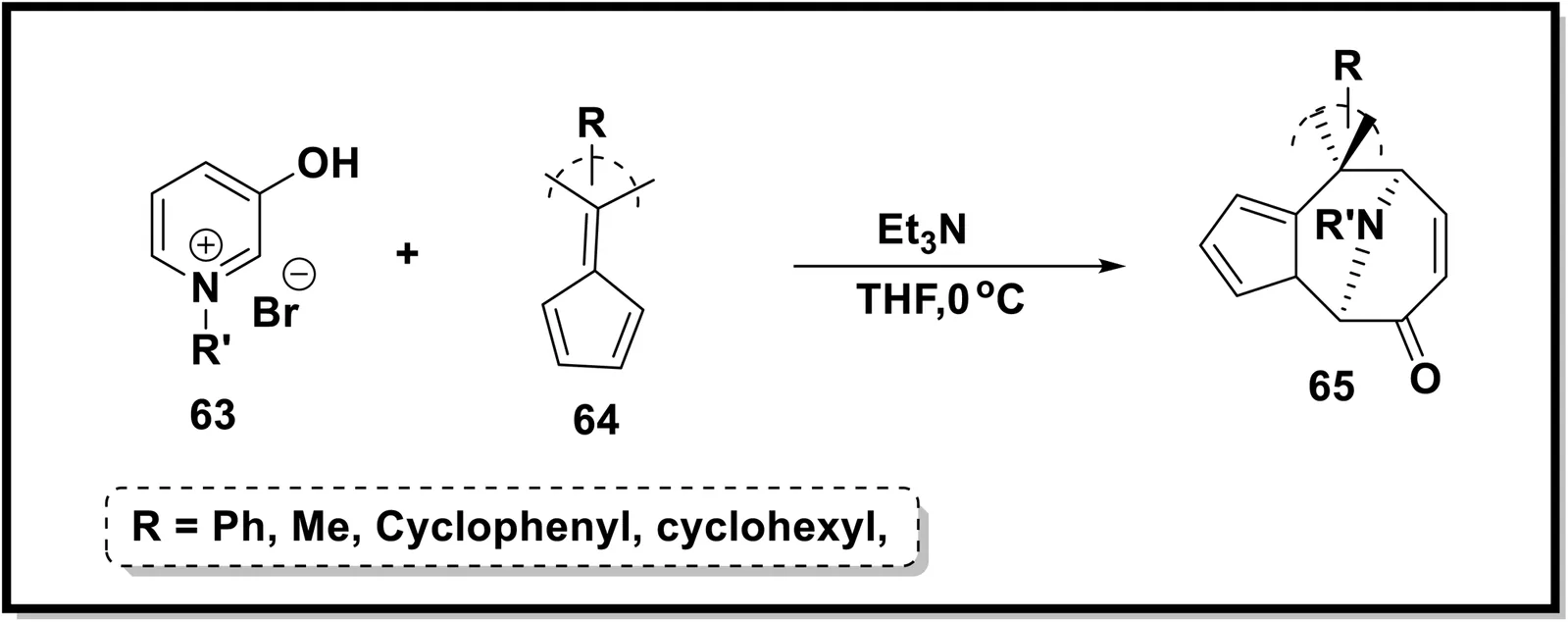
[6 + 3] CR promoted the synthesis of bicyclo [6.3.0] undecane derivatives.^40^

Further, his team also explored the triethylamine-catalysed [6 + 3] cycloadditions involving the 3-oxidopyridinium betaines 63 with pentafulvenes 64 (Fig. 22) for the synthesis of bicyclo [6.3.0] undecane derivatives 65 (68%).^40^

**Fig. 23:**
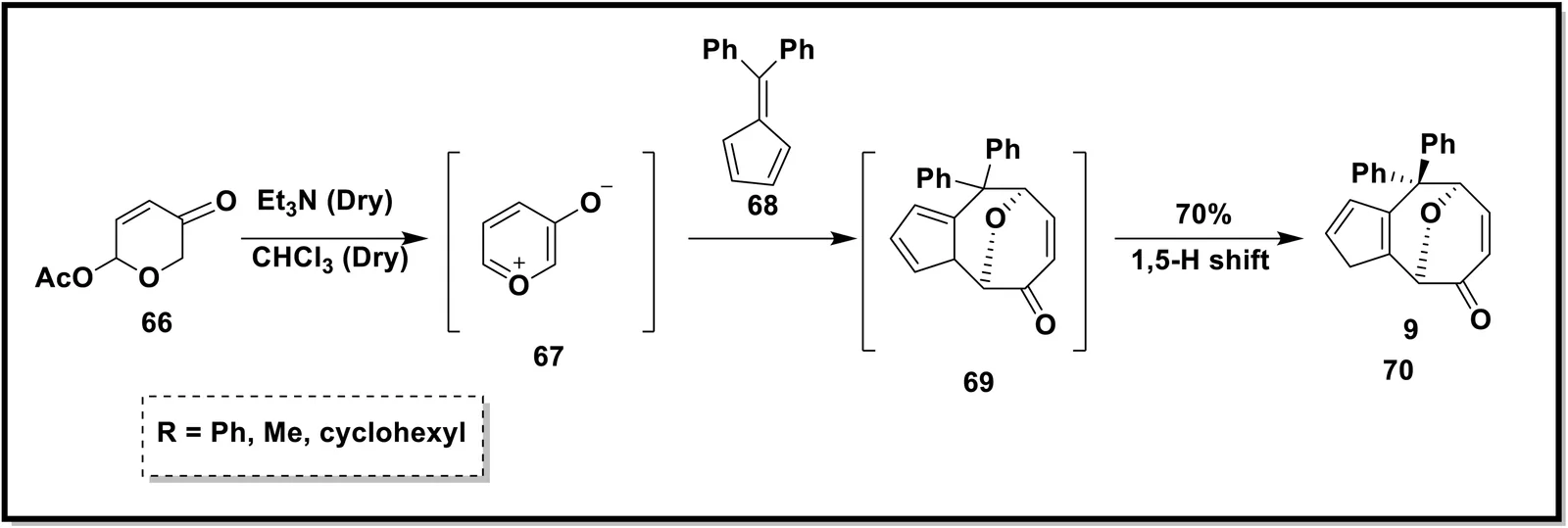
The reaction pathway for the synthesis of oxabridged cyclooctanoids using [6 + 3] CR.^41^

Krishnan *et al.* have explored the [6 + 3] cycloadditions of pentafulvenes 68 with 3-oxidopyrylium betaine 66, for the *in situ* production of pyranulose acetates 69, which further led to the regioselective formation of 5–8 fused oxabridged cyclooctanoids 70 (Fig. 23). Triethylamine as the preferred base, and dry chloroform at 50 °C were the best reaction conditions for the production of 5–8 fused oxabridged cyclooctanoids 70. The cycloaddition proceeded efficiently with a broad scope of fulvenes 6,6-diaryl, 6,6-dimethyl, cycloalkyl and 6-aryl-substituted and afforded functionalized oxabridged cyclooctanoids 70 in moderate to excellent yields (45–83%) with complete regioselectivity. This approach offered an efficient route to eight-membered oxygen-bridged frameworks, which have immense relevance in natural products.^41^

**Fig. 24:**
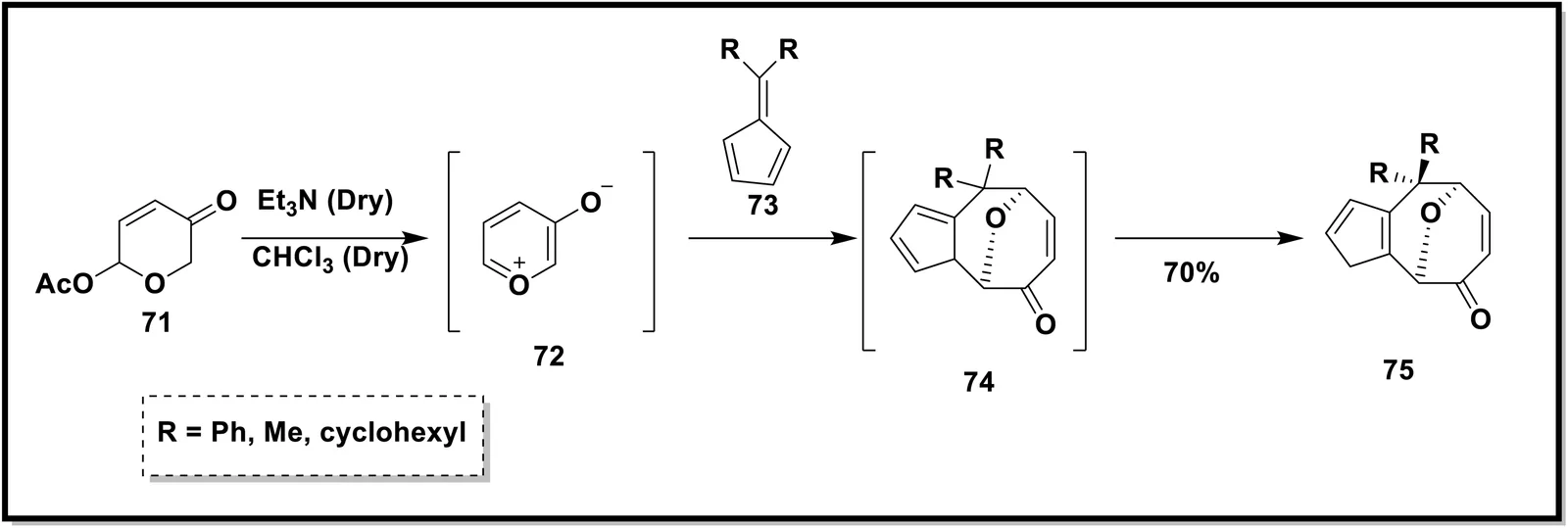
The synthesis of cyclooctanoid scaffold *via* [6 + 3] CR.^42^

Radhakrishnan *et al.* reported a formal [6 + 3] cycloaddition to synthesize fused cyclooctanoids 75 using fulvenes 73 with 3-oxidopyrylium betaines 71 (Fig. 24). The treatment of 3-oxidopyrylium betaine 71 with 6,6-diphenylfulvene 73 under the model conditions delivered the [6 + 3] cycloadduct in 70% yield. Various fulvenes reacted efficiently under optimized conditions (Et_3_N, CHCl_3_, 50 °C) to afford fused oxabridged cyclooctanoids in 57–83% yield.^42^

**Fig. 25:**
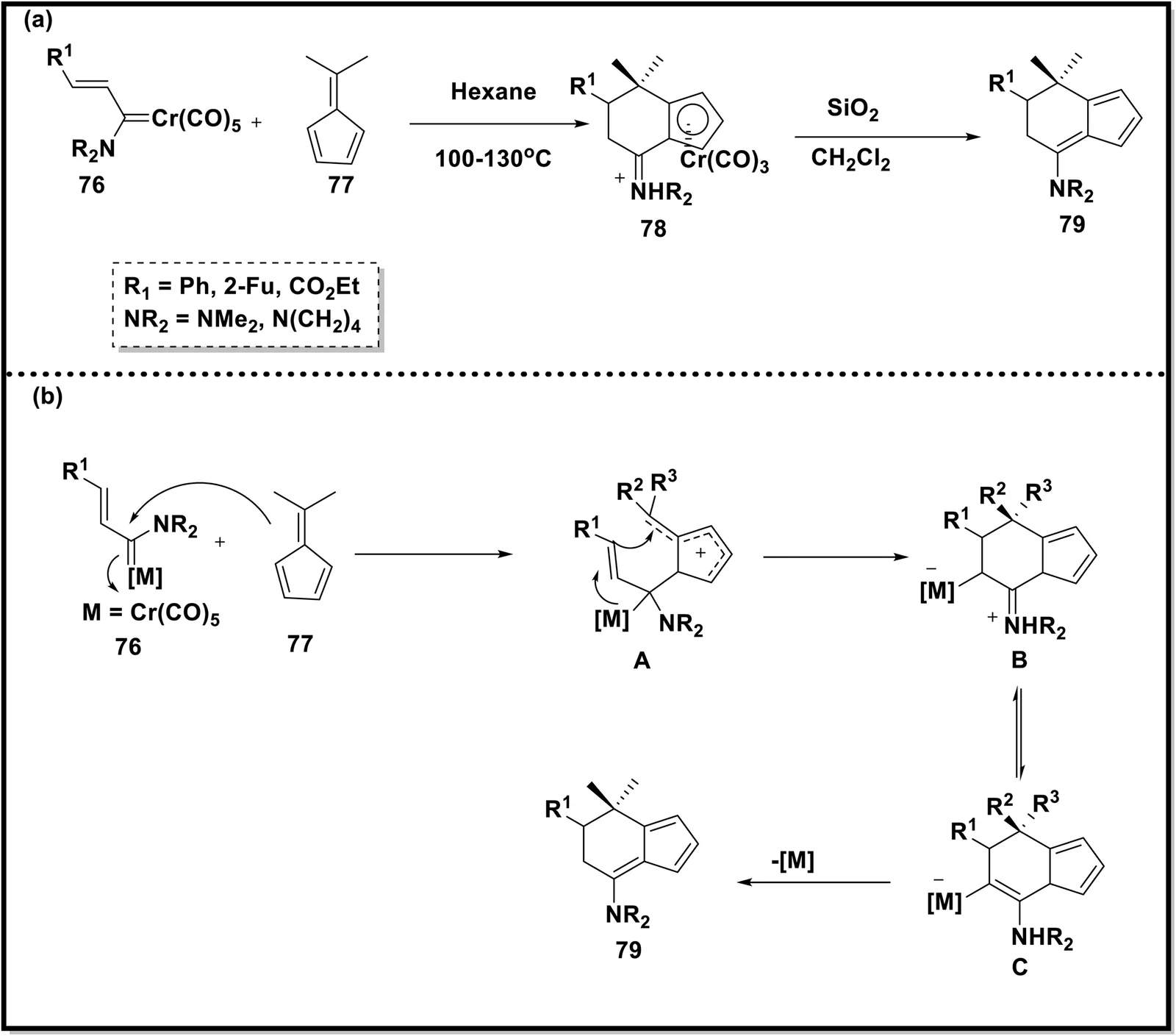
(a) Synthesis of indene derivatives *via* [6 + 3] cycloaddition; (b) the reaction mechanism of [6 + 3] CR for the synthesis of indene derivatives.^43^

Barluenga *et al.* described the [6 + 3] cycloaddition reaction for the synthesis of the dihydroindene and indene derivatives. The methodology involved the [6 + 3] cycloaddition of Fischer alkenyl(aminocarbene) complexes with pentafulvenes (Fig. 25a). The reaction of aminocarbenes 76 with 6,6-dimethylfulvene 77 resulted in the formation of tricarbonyl–dihydroindene complexes 78, in good yields (80–90%).^43^

The mechanistic path of the reaction is outlined in Fig. 25b. The reaction begins with the 1,2-nucleophilic addition of fulvene 77 to the electrophilic carbene carbon of the Fischer aminocarbene chromium complex 76, leading to the zwitterionic intermediate A. Further, intermediate A undergoes a [1,2]-migration of the Cr(CO)_5_ fragment, followed by intramolecular cyclization to form intermediate B. Subsequently, intermediate B generates the enamine intermediate C through consecutive metal elimination and *η*^2^-olefin coordination. Finally, intermediate C undergoes either isomerization to furnish the stable tricarbonyl(dihydroindene)chromium complexes or metal decoordination followed by tautomerization/aromatization to afford the indene derivatives 79.

**Fig. 26:**
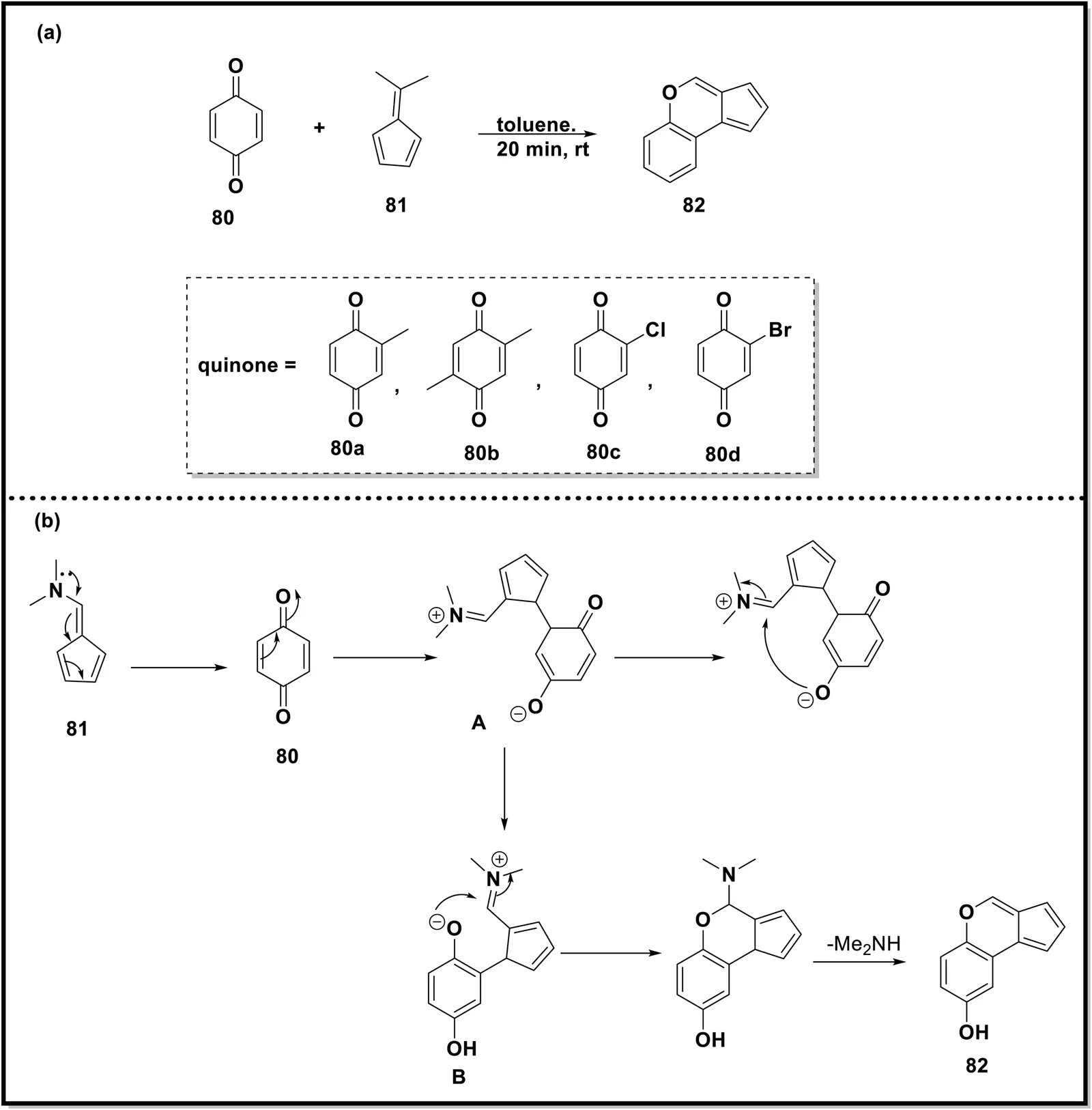
(a) A synthesis of cyclopenta[*c*]-fused heterocyclic frameworks *via* [6 + 3] CR; (b) a mechanistic pathway for the synthesis of cyclopenta[*c*]-fused heterocyclic compounds.^44^

Hong and co-workers developed a hetero [6 + 3] cycloaddition between 6-dimethylaminofulvene 81 and benzoquinones 80, enabling efficient access to cyclopenta[*c*]-fused heterocyclic frameworks 82 (Fig. 26a). The reaction proceeded smoothly under metal-free conditions by simply stirring the reactants in dry benzene at ambient temperature, highlighting the protocol's operational simplicity. Among the fulvene substrates examined, 6-dimethylaminofulvene was essential, as the increased π-electron density directed the chemoselectivity toward the [6 + 3] pathway rather than the conventional [4 + 2] cycloaddition. Solvent screening revealed that benzene was optimal, affording the desired hetero [6 + 3] cycloadduct as the sole isolable product. Under the optimized conditions, the cycloaddition furnished cyclopenta[*c*]chromene derivatives 82 in good yields (up to 65%) with complete regioselectivity. The study further demonstrated that the reaction tolerates a range of benzoquinone substituents, underscoring the robustness of the [6 + 3] cycloaddition strategy for constructing complex polycyclic heterocycles.^44^

The mechanistic pathway of the reaction is showcased in Fig. 26b. The initial nucleophilic addition of electron-rich 6-dimethylaminofulvene 81 to benzoquinone 80, generates the zwitterionic intermediate A. Subsequently, intermediate A undergoes tautomerization to produce the hydroquinone intermediate B, which further undergoes intramolecular *O*-cyclization followed by elimination of dimethylamine to furnish the cyclopenta[*c*]chromene product 82.

#### 2.2.2. Lewis acid-catalyzed [6 + 3] cycloaddition and formal [6 + 3] annulation reactions

**Fig. 27:**
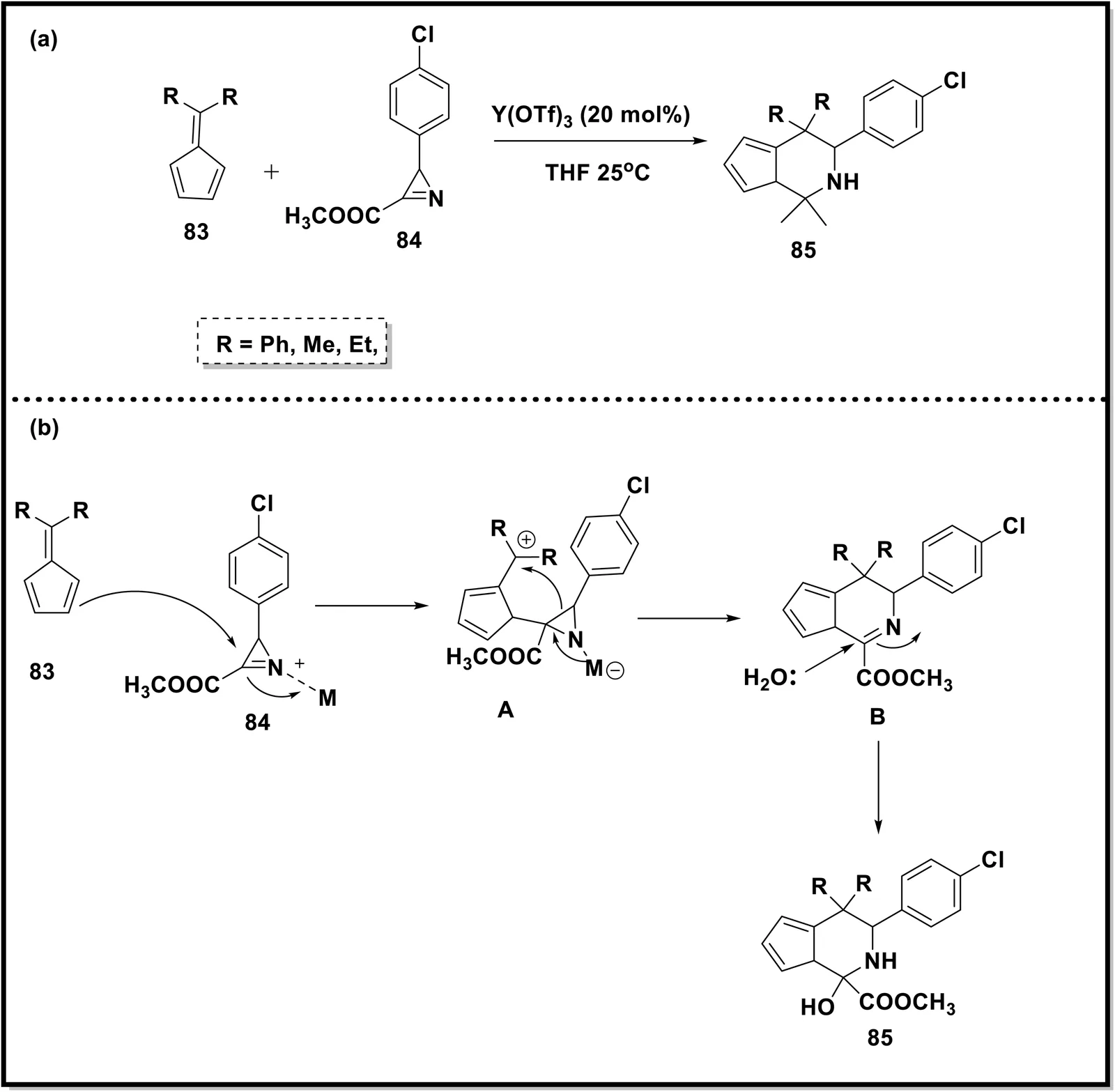
(a) Synthesis of pyridine derivatives *via* [6 + 3] cycloadditions; (b) a mechanistic pathway for the synthesis of pyridine derivatives.^45^

Hong and co-workers have also explored the [6 + 3] cycloadditions of fulvenes 83 and 2*H*-azirines 84 to afford pyrindine derivatives 85 (Fig. 27). The optimization showed that introducing Y(OTf)_3_ as a Lewis acid markedly improved the reaction outcome, affording the cycloadduct in good yield (83%). Further, the reaction with triethylamine in dichloromethane induced rearrangement to stable isomers without compromising regioselectivity. The comprehensive screening of fulvene substrates revealed wide applicability and delivered [2]pyrindines in good yields (up to 85%). This approach provides an efficient strategy for constructing [2]pyrindine framework motifs commonly found in natural products.^45^

The mechanistic pathway is outlined in Fig. 27a. Initially, Lewis acid-activated 2*H*-azirine 84 undergoes nucleophilic attack by fulvene 83 to generate zwitterionic intermediate A through a stepwise process. Subsequently, intermediate A undergoes ring opening and intramolecular cyclization to furnish the fused [2]pyrindine intermediate B. Further base-mediated isomerization of B affords the thermodynamically stable pyrindine derivative 85.^45^

All papers were also summarized in Table 2.

**Table 2:** Summary of [6 + 3] cycloaddition reactions (2000–2026)

S. no.	6C-synthon	3C-synthon	Product	Condition	Yield/enantioselectivity/diatereoselectivity	Author^a^/year
**Metal catalysts**						
*Palladium-catalyzed*						
1	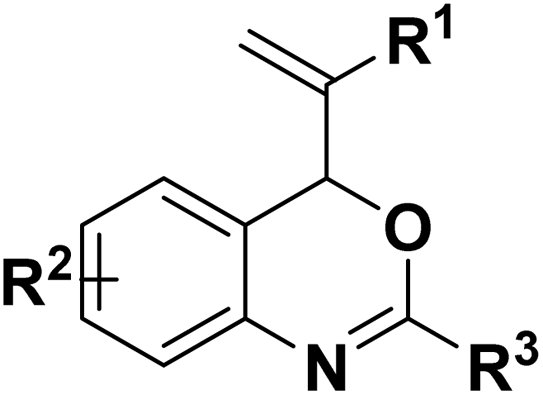	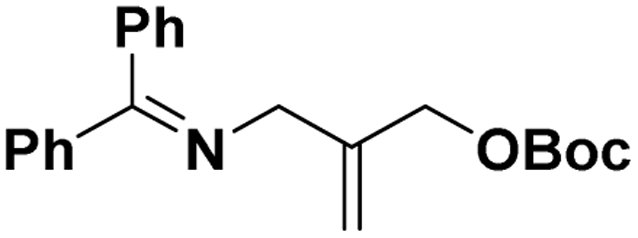	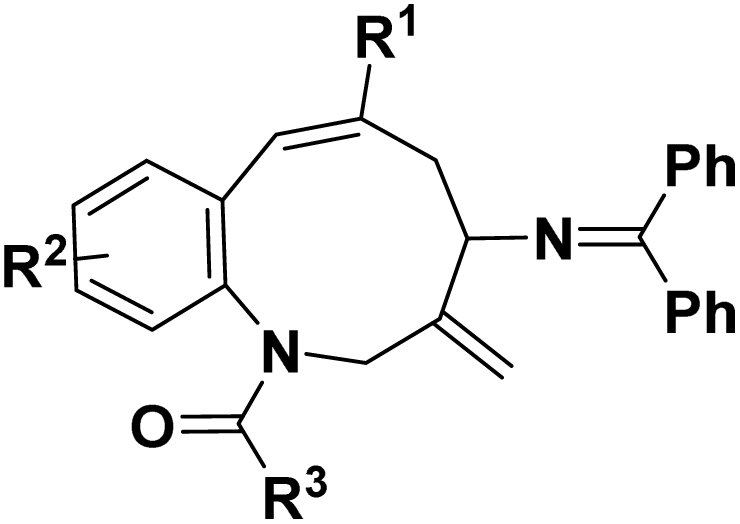	Ar atmosphere, Pd_2_(dba)_3_ (5 mol%)	60–75%/77–93% ee	Chen *et al.*^22^/2026
				A1 (20 mol%)		
				DCM (1.0 mL), rt, 24 h		
2	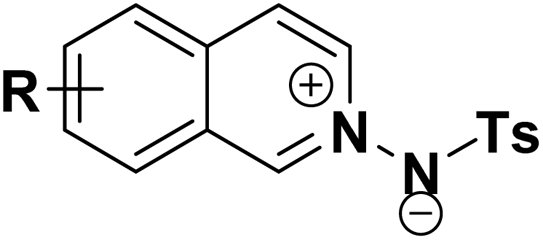	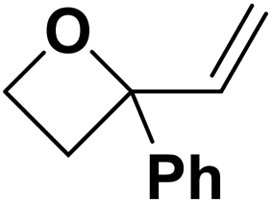	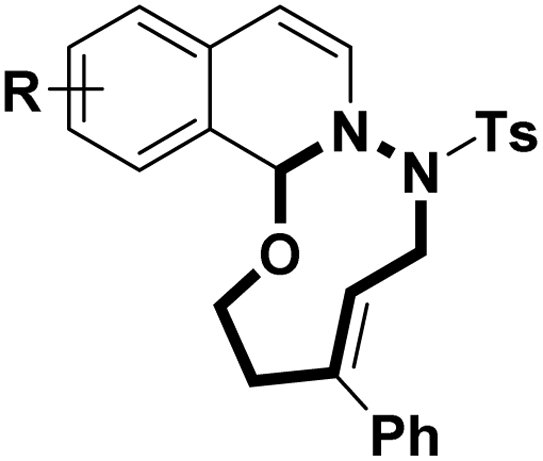	Pd(PPh_3_)_4_ (3.0 mol%)	90–98%	Xie *et al.*^21^/2022
				PPh_3_ (6.0 mol%)		
				DCM, rt		
3	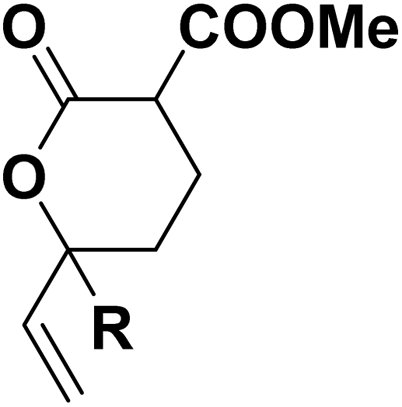	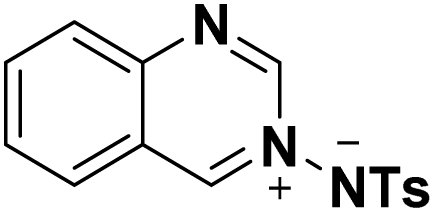	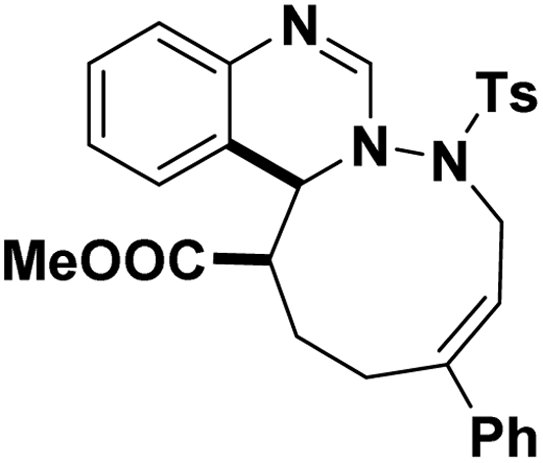	Pd_2_dba_3_·CHCl_3_ (5 mol%)	94–98%, >20 : 1 d.r.	Li *et al.*^23^/2022
				B1 (20 mol%) DCM, 25 °C		
4	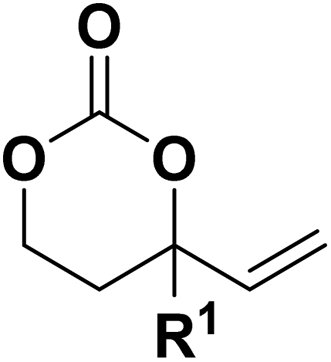	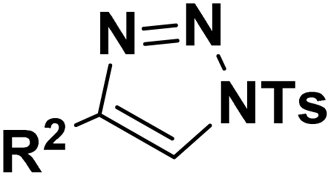	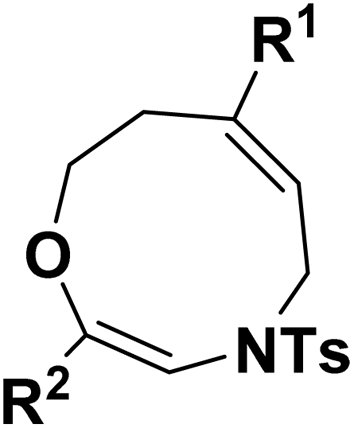	PdCp(allyl) (5.0 mol%)	70–80%	Lee *et al.*^24^/2021
				C1 (10.0 mol%)		
				Rh_2_esp_2_ (2.5 mol%) toluene, 60 °C		
5	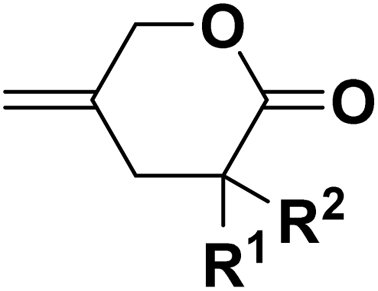	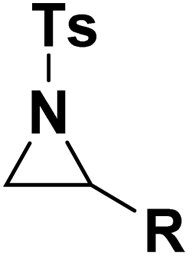	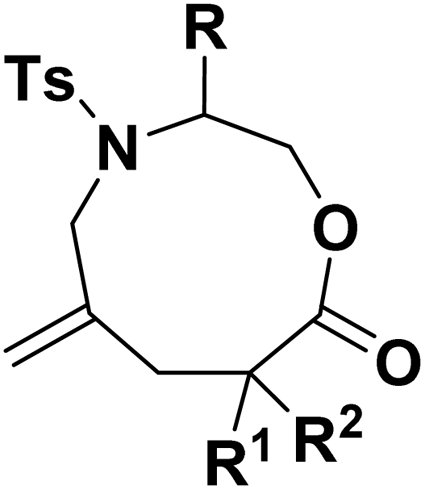	PdCp(–C_3_H_5_)	53–87%	Shintani *et al.*^25^/2011
				(5mol%), D1 toluene ,65 °C, 48 h		
6	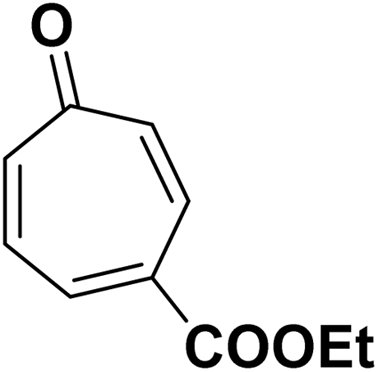	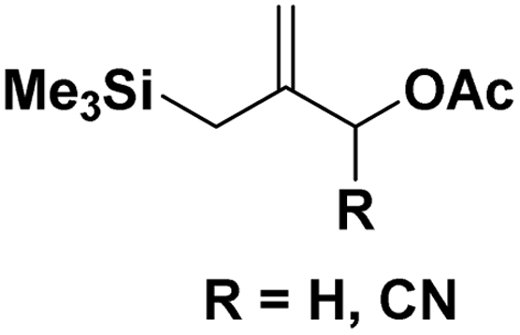	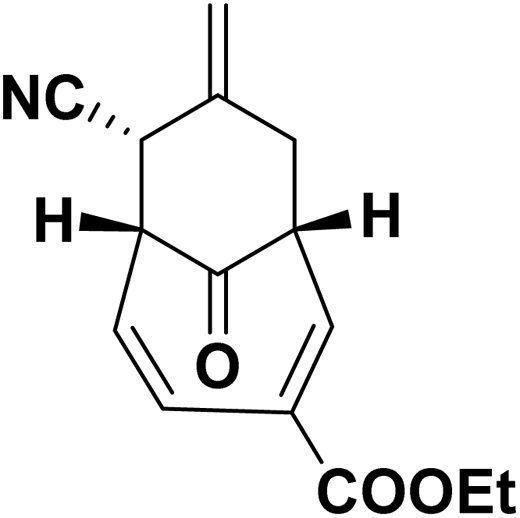	Pd(dba)_2_ (5 mol%)	64–94%/86–99% ee >10 : 1 d.r.	Trost *et al.*^26^/2009
				E1 (10 mol%)		
				15 h		
7	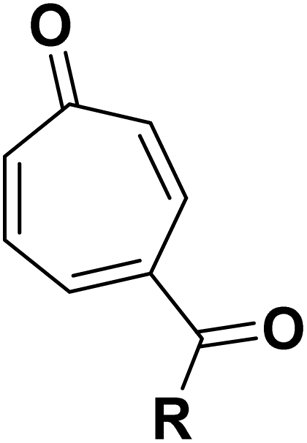	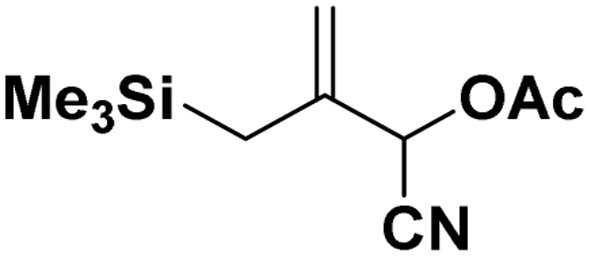	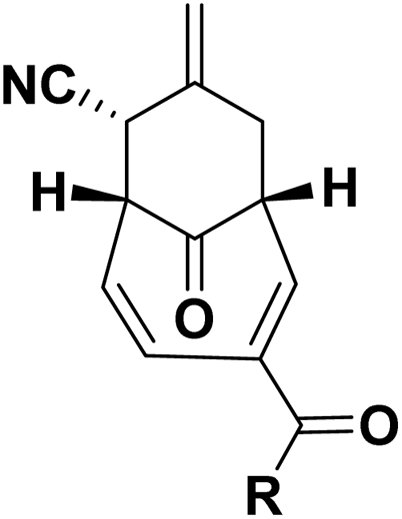	Pd(dba)_2_ (5 mol%)	64–94%, 86–99% ee	Trost *et al.*^27^/2008
				E1 (10 mol%)		
				15 h		
*Copper-catalyzed*						
8	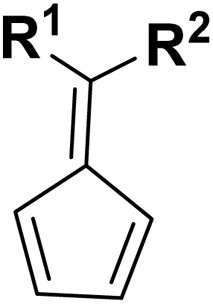	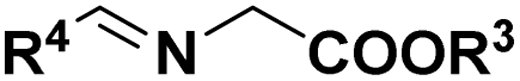	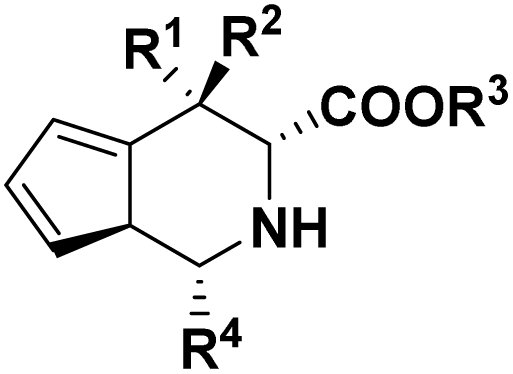	Cu(MeCN)_4_	74–78% 83–94% ee)	Tao *et al.*^28^/2013
				BF_4_-(*S*)-F1		
				Cs_2_CO_3_, CH_2_Cl_2_		
9	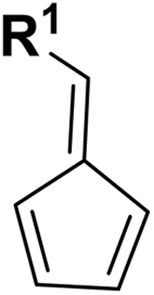	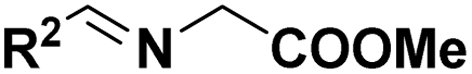	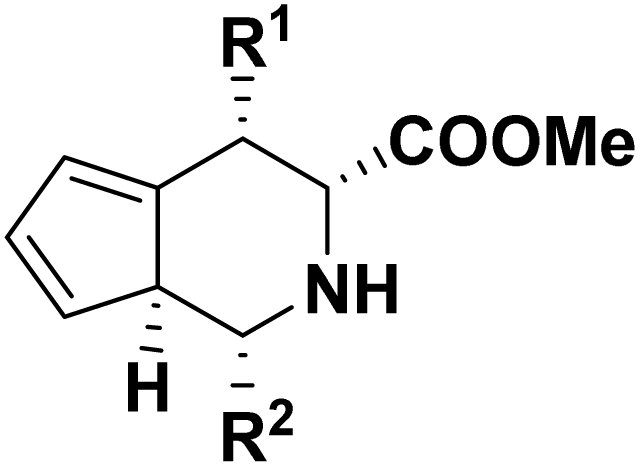	Cu(i)/H1	46–96%, d.r. 81, 90% ee (*endo*)	Narayan *et al.*^29^/2014
				Et_3_N (1 eq.) dioxane, rt, 0.5 h (*endo*)	25–86%, d.r. 80, 93% ee (*exo*)	
				Cu(i)/G1		
				Et_3_N (1 eq.)		
				THF, rt, 0.5 h(*exo*)		
10	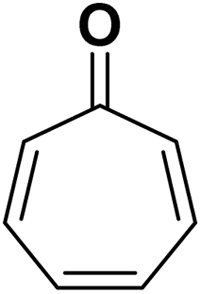	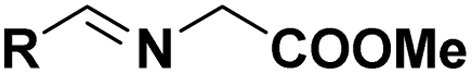	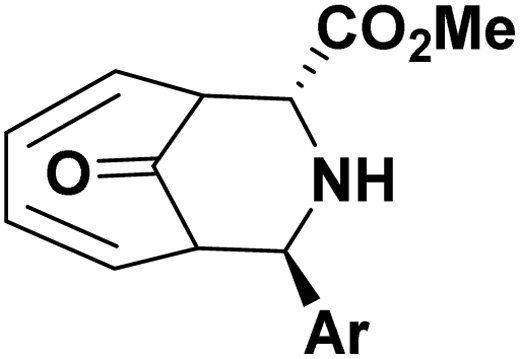	Cu(i)/I1 (5 mol%)Et_3_N (15 mol%), DCM 18–24 h	80–85%, >20 : 1 d.r.	Teng *et al.*^30^/2014
11	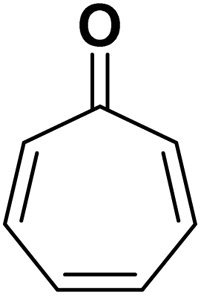	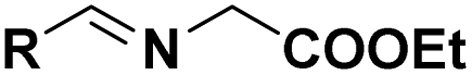	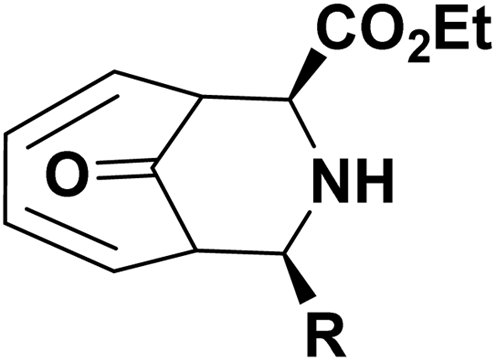	Cu(CH_3_CN)_4_ClO_4_/J1	85–91%, >20 : 1 d.r.	Liu *et al.*^31^/2014
				DBU, DCM, 6 h		
12	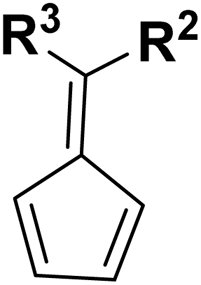	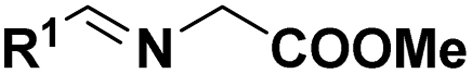	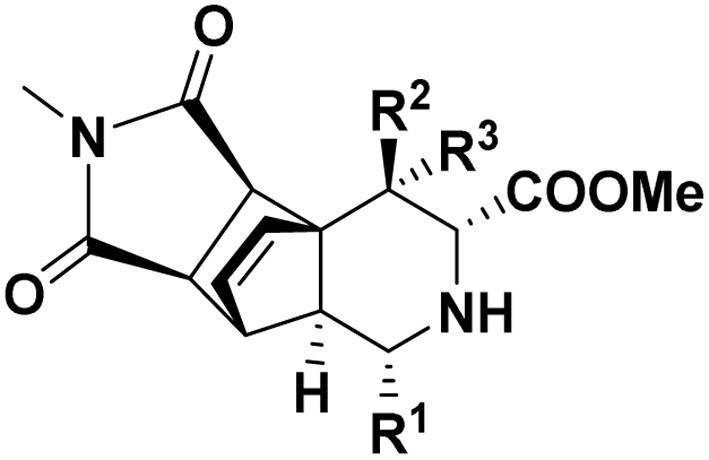	Cu(CH_3_CN)_4_	76–81%, 95% ee, 85 : 15 d.r.	Potowski *et al.*^32^/2013
				BF_4_/K1		
				Et_3_N (1 eq.)		
				THF, −40 °C, 3–12 h		
13	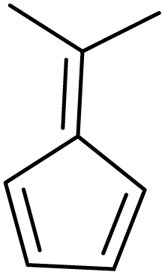	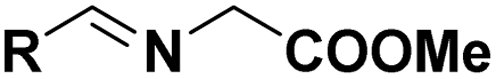	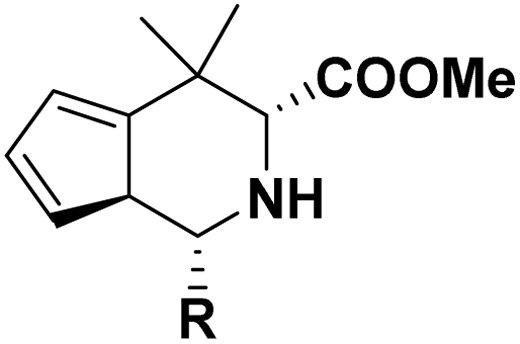	[Cu(CH_3_CN)_4_]	75–80%	Potowski *et al.*^33^/2012
				BF_4_, L1		
				Cs_2_CO_3_, CH_2_Cl_2_		
14	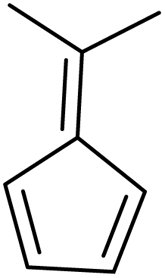	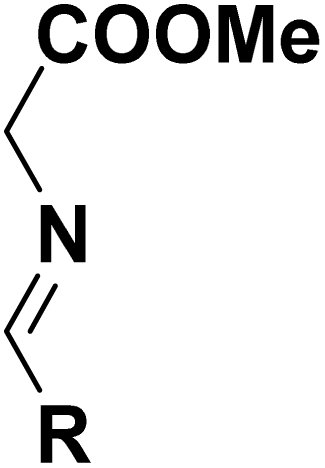	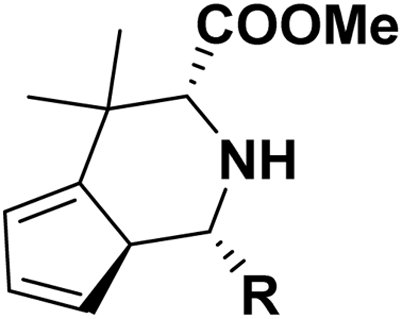	CuBF_4_/M1(5 mol%)Cs_2_CO_3_, CH_2_Cl_2_,−40 °C	87%, 99% ee	He *et al.*^34^/2012
*Silver-catalyzed*						
15	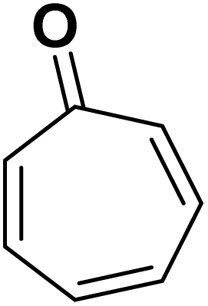	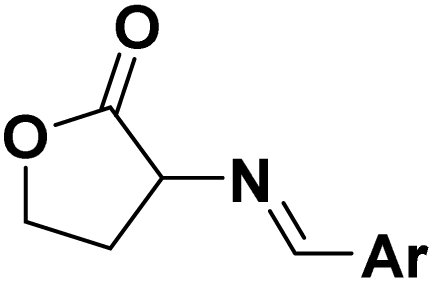	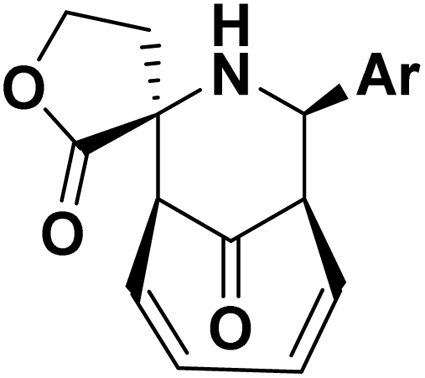	AgOAc (10 mol%)	92%	Wu *et al.*^35^/2016
				PPh_3_ (20 mol%)		
				DBU (20 mol%)		
				MeOH, rt		
16	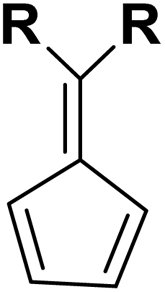	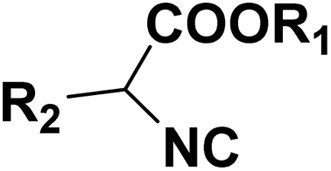	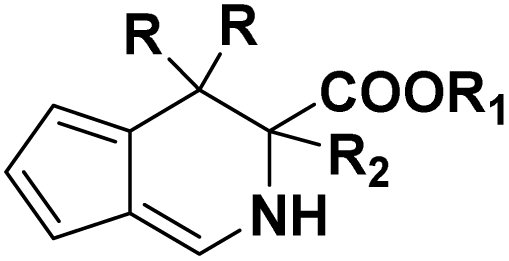	AgOAc/PPh_3_(3.0 mol%) Et_3_N (15 mol%)	95%	He *et al.*^36^/2015
				DCM, rt		
17	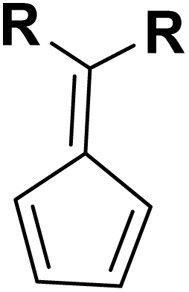	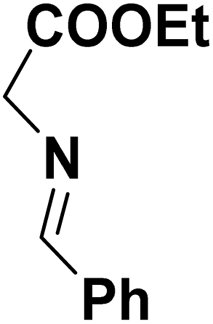	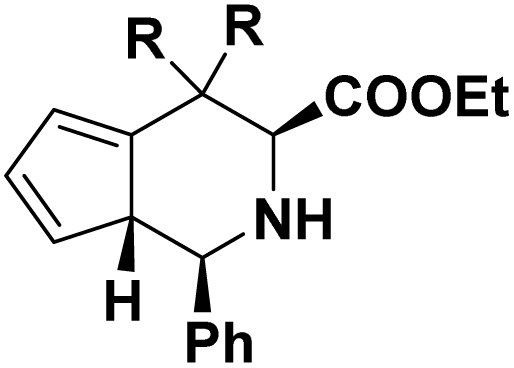	Ag_2_O, Et_3_N	92%	Hong *et al.*^37^/2003
				THF, 25 °C		
*Chromium-catalyzed*						
18	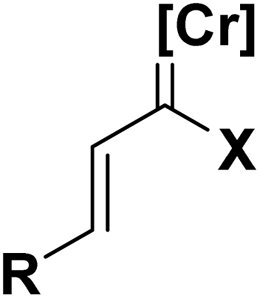	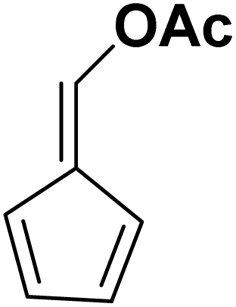	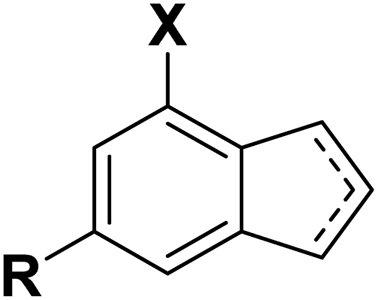	MeCN, 80 °C or toluene	69–75%	Barluenga *et al.*^38^/2001
				100–200 °C SiO_2_, CH_2_Cl_2_		
**Non-metal catalysts**						
*Organocatalysts*						
19	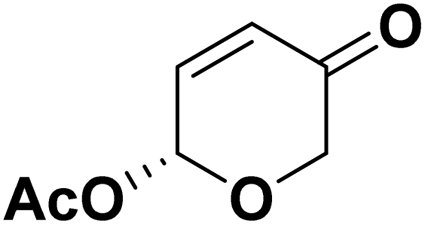	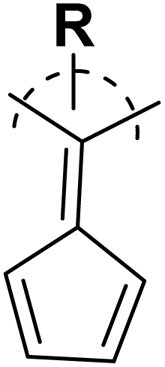	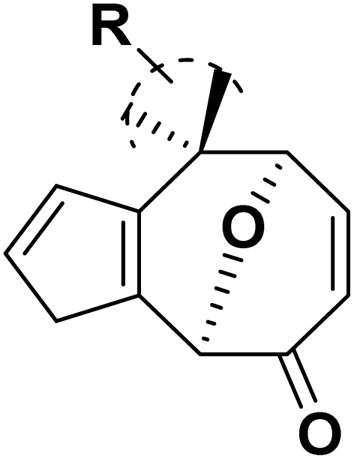	Et_3_N, CHCl_3_ 50%	72–83%	Kuthanapillil *et al.*^39^/2013
				6 h, 68%		
20	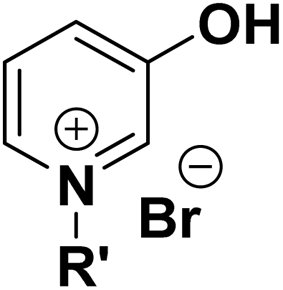	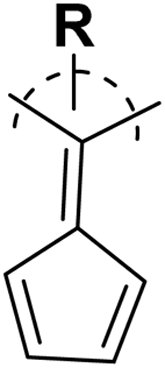	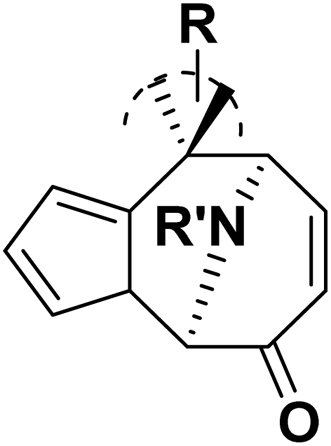	Et_3_N, THF, 0 °C	66–72%	Kuthanapill *et al.*^40^/2011
21	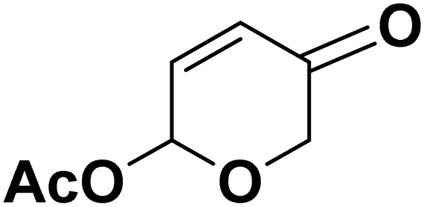	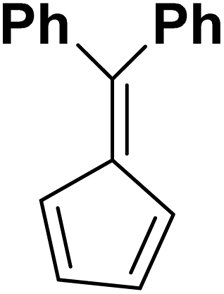	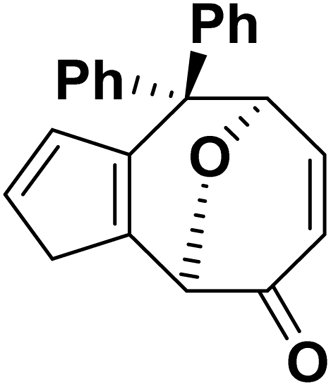	Et_3_N (dry), CHCl_3_(dry) 50%	79–83%	Krishnan *et al.*^41^/2006
22	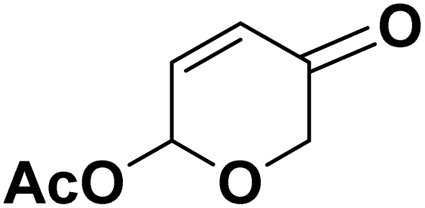	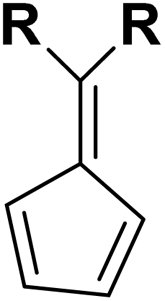	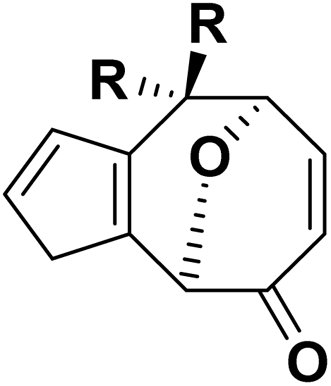	Et_3_N (dry)	57–83%	Radhakrishnan *et al.*^42^/2005
				CHCl_3_(dry) 50%		
23	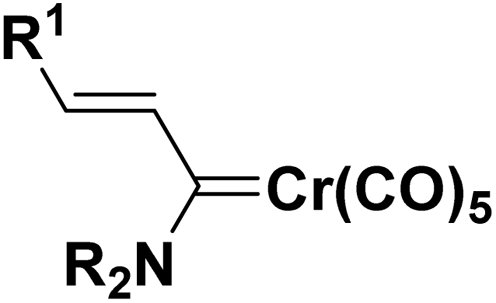	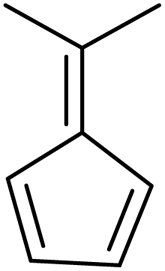	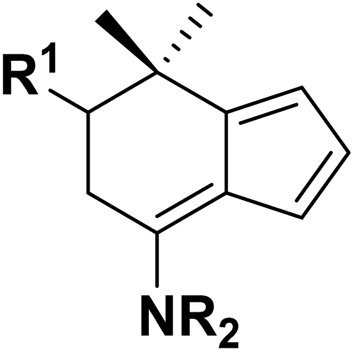	Hexane 100–130 °C, SiO_2_ CH_2_Cl_2_	90–100%	Barluenga *et al.*^43^/2005
24	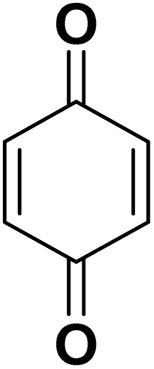	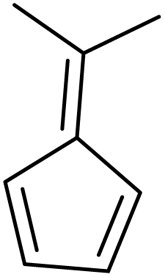	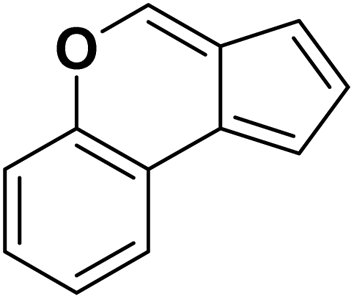	Toluene	76–80%	Hong *et al.*^44^/2005
				20 min, rt		
*Lewis-acid catalyst*						
25	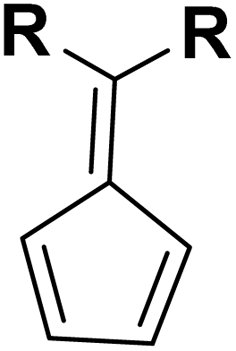	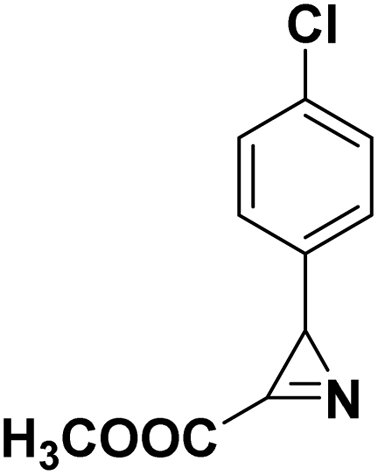	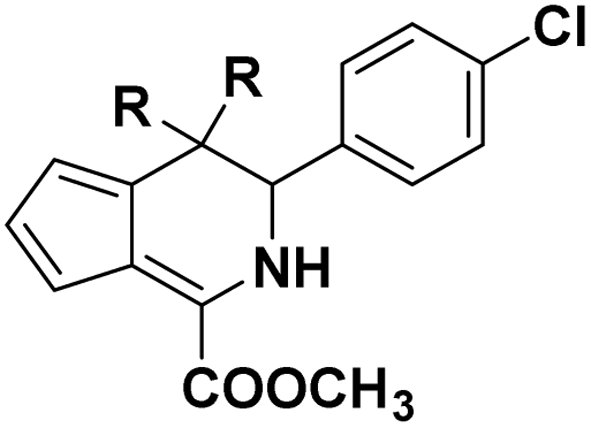	Y(OTf)_3_	93–100%	Hong *et al.*^45^/2004
				THF 25 °C		

a References.

## 3. Summary and outlook

The [6 + 3] cycloaddition and [6 + 3] annulation are powerful synthetic methodology that enables the construction of nine-membered carbocyclic, heterocyclic, fused, and bridged cyclic frameworks through the union of a 6-atom π-system and a 3-atom synthon. [6 + 3] cycloadditions provide direct access to medium-sized rings, which are usually challenging to synthesize using conventional approaches. This methodology consistently demonstrates high levels of regio- and stereocontrol, making it particularly attractive for complex ring synthesis.

In recent years, significant progress in transition-metal catalysis has further enhanced its synthetic utility. Catalytic systems based on palladium, chromium, copper, and silver have been extensively explored and shown to deliver excellent regioselectivity, diastereoselectivity, and enantioselectivity. These developments have considerably expanded the structural diversity accessible through this strategy and facilitated the synthesis of biologically important molecular architectures.

In addition to transition-metal catalysis, organocatalytic strategies have emerged as valuable alternatives, further broadening the scope and versatility of [6 + 3] cycloaddition reactions. Recent reports demonstrate that a diverse array of substrates, including vinylvalerolactones, trimethylenemethane derivatives, isocyanoacetates, azomethine ylides, and tropones can be effectively engaged under organocatalytic conditions. These studies collectively underscore the growing maturity of organocatalytic approaches in medium-ring synthesis.

## 4. Outlook

Despite substantial progress, [6 + 3] cycloaddition chemistry remains comparatively underdeveloped relative to other cycloaddition strategies. Several promising directions are anticipated to shape future research, such as exploration of unconventional 6π and 3π-carbon synthons, use of more efficient reagents compatible with a broader range of functional groups and heteroatom-containing systems. Further, the utilization of visible-light photoredox catalysis and electrochemical activation needs to be explored in these [6 + 3] cycloadditions. Moreover, the application of density functional theory (DFT) and machine learning tools to predict reactivity, selectivity, and catalyst performance needs to be evaluated for the [6 + 3] cycloadditions. Continued advances in catalysis, asymmetric synthesis, photochemistry, and computational chemistry are anticipated to further expand its synthetic utility. With new anticipated discoveries of catalytic platforms and reaction partners, the [6 + 3] cycloaddition chemistry is poised to become an increasingly important tool in modern organic synthesis, natural product construction, and pharmaceutical development.

## Conflicts of interest

The authors declare no conflict of interest.

## Data Availability

Data sharing does not apply to this article, as no new data were created or analyzed in this study.
